# Recognition-dependent activation of the *Arabidopsis* RRS1-R/RPS4 TIR-NLR immune receptor pair

**DOI:** 10.1073/pnas.2611802123

**Published:** 2026-10-27

**Authors:** Hee-Kyung Ahn, Guanghao Guo, Jan Skłenar, Shania Pin Yin Keh, Sung Un Huh, Michelle T. Hulin, Hayden Burdett, Maria Sindalovskaya, Seungjin Na, Jihyeon Choi, Nitika Mukhi, He Zhao, Lena S. Knorr, Eunok Paek, Mark J. Banfield, Frank L. H. Menke, Wenbo Ma, Jonathan D. G. Jones

**Affiliations:** ^a^ School of Biological Sciences, School of Biological Sciences, Institute of Molecular Plant Sciences, University of Edinburgh, Edinburgh EH9 3BF, United Kingdom; ^b^ The Sainsbury Laboratory, University of East Anglia, Norwich Research Park, Norwich NR4 7UH, United Kingdom; ^c^ Department of Biological Science, Kunsan National University, Gunsan-si 54150, Republic of Korea; ^d^ Department of Plant Soil and Microbial Sciences, Michigan State University, East Lansing, MI 48824; ^e^ Department of Biochemistry and Metabolism, John Innes Centre, Norwich Research Park, Norwich NR4 7UH, United Kingdom; ^f^ Center for Target-to-Therapeutics Research, Korea Basic Science Institute, Cheongju 28119, Republic of Korea; ^g^Convergent Analytical Science, University of Science and Technology, Daejeon 34113, Republic of Korea; ^h^ Department of Computer Science, Hanyang University, Seoul 04763, Republic of Korea

**Keywords:** plant immunity, TIR domain, paired NLRs, NLR, NADase

## Abstract

Plant TIR domain-containing proteins exhibit NADase activity upon pathogen effector recognition, creating small molecules that activate defense via EDS1 and its associated proteins. *Arabidopsis* RPS4 and RRS1 comprise a TIR-NLR immune receptor pair that recognizes multiple effector proteins including AvrRps4 and PopP2. We show here that RPS4 and RRS1 acquire NADase activity upon recognition of a cognate effector. However, both in the presence and absence of effector, RPS4 and RRS1 form an oligomer that does not change in size upon effector provision. Effector-dependent defense activation by RPS4 and RRS1 requires the catalytic glutamate residue and the BB-loop motif of RPS4, which is absent in RRS1 and other RRS1-like TIR domains in *Arabidopsis*. Oligomerization of RPS4 and RRS1 occurs through multiple domains, but oligomerization does not require enzymatic activity. We propose that conformational changes or reconfiguration within the RPS4/RRS1 oligomer upon effector recognition derepresses the complex for immune activation.

## Significance

TIR domains that are conserved across several kingdoms of life have NAD^+^ -hydrolyzing activity that activates immune signaling. Most of the TIR domains identified to date which exhibit NADase activity assemble into homomers. We show here that the *Arabidopsis* paired TIR-NLRs RRS1-R and RPS4 assemble in a heteromeric protein complex in the preactivation state. NADase activity of purified RRS1-R/RPS4 protein complex is only observed upon effector-dependent activation, and not in the absence of effector, suggesting conformational change of the complex in an effector-dependent manner leads to NADase activity. The RRS1-R TIR domain lacks conserved motifs and residues required for NADase activity. The lack of NADase activity in RRS1-R TIR domain may inhibit autoactivation due to preformation of the complex.

TIR protein domains are highly conserved across taxonomic kingdoms (1). Self-association of TIR domains leads to new functions, either bringing together different proteins as a scaffold or generating NADase activity as an enzyme (2). NADase activity from TIR domains is common in prokaryotes and plants, and vertebrate TIR-domain containing SARM1 also has NADase activity (3). In plants, TIR domains are usually the N-terminal domain of intracellular NLR (nucleotide-binding, Leucine-rich repeat) immune receptors (4). Self-association of these TIR-NLRs upon effector recognition leads to NADase activity (5–7), which subsequently results in localized cell death, or hypersensitive response (HR).

Structural analysis of activated plant TIR-NLRs revealed four TIR-NLR protomers each bound to their cognate effector (8–11). In these structures resolved by cryo-EM, TIR domains are assembled into a “dimer of dimer” configuration, creating two NADase active sites from each TIR-domain dimer. Mammalian and bacterial TIR domains can also show NADase activity, with distinct conformations from a dimer of dimer form (2). However, in all cases, the catalytic glutamate residue (5, 6) and aspartate and glycine residues in the BB-loop region are crucial for NADase activity (12). At present, there are no examples of heteromeric TIR domain-containing proteins that show NADase activity.

Some NLRs require other NLRs for function (13). Paired NLR genes can often be identified in the genome by their proximity to each other and their divergent transcription (14). Paired NLR proteins can be classified into “sensor” NLRs and “executor/helper” NLRs, with the sensor component often having an integrated domain (ID) that senses effector molecules. The executor NLR often displays autoactivity upon overexpression, which can be suppressed by coexpression of the sensor NLR (15). However, for some paired NLRs, expression of executor NLR without sensor NLR does not induce autoactivity (16).

Dimerization of TIR domains is mediated by the AE interface, where the histidine ring on one protomer packs with the histidine ring on the other (8–11, 17). This association is supported by the serine or glycine residues nearby (17). The AE interface was defined from structural analysis of a heteromeric interaction between the TIR domains of the *Arabidopsis* RPS4 and RRS1 paired NLRs (17). AE interface mutations prevent the interaction between RRS1-R and RPS4 (17). The AE interface is critical for interaction between another *Arabidopsis* paired TIR-NLR CHS3/CSA1 (18). While the AE interface is required for repression of autoactivity by RPS4, the same AE interface is required for effector-dependent activation (17). How the same interface functions to repress and derepress is unknown.

*Arabidopsis* RRS1-R (Resistance to *Ralstonia solanacearum* 1; sensor) and RPS4 (Resistance to *Pseudomonas syringae* 4; executor) comprise a TIR-NLR pair that has been extensively studied as a model for paired NLRs. The RRS1-R allele from Ws-2 accession confers recognition of both AvrRps4 and PopP2, whereas the Col-0 RRS1-S allele lacks 83 amino acids at the C-terminus compared to RRS1-R and confers only AvrRps4 recognition. We previously reported the repression of RPS4 autoactivation by RRS1-R (15, 17), as well as derepression of RPS4/RRS1-R upon effector recognition (19). RRS1-R and RPS4 recognize multiple effectors from diverse pathogen species; AvrRps4 from leaf pathogenic bacteria *P. syringae pv. pisi* (20), PopP2 from root pathogenic bacteria *R. solanacearum* (21), XopJ6 from *Xanthomonas campestris* which causes black rot in *Brassicaceae* (22), and unknown effector(s) from fungal pathogen *Colletotrichum higginsianum* (23, 24). AvrRps4 is recognized by RRS1-R through interaction with the integrated WRKY domain (25), while PopP2 and XopJ6 are acetyltransferases; the resulting WRKY acetylation activates the RRS1-R/RPS4 complex, whereas catalytic mutant PopP2 C321A fails to acetylate RRS1-R WRKY domain (26, 27). Modification of the WRKY domain of RRS1-R leads to an increase in intramolecular affinity between the RRS1-R TIR domain and the C-terminal IDR (intrinsically disordered region) domain (28). Effector recognition leads to changes in proximity between TIR domains of RRS1-R and RPS4 (TIR^RRS1^ and TIR^RPS4^, respectively) while maintaining RRS1-R/RPS4 interaction and protein abundance. Phosphorylation of the C-terminal domains of RRS1-R is essential for PopP2 recognition, but not AvrRps4 (28).

We report here that the purified *Arabidopsis* RRS1-R/RPS4 complex hydrolyzes NAD^+^ only when coexpressed with the cognate effector. Despite the activity change, the RRS1-R/RPS4 complex does not change in size on nondenaturing, blue-native PAGE (BN-PAGE) analysis after effector recognition. These and our previous data (19, 28) suggest that the activated complex likely involves a reconfiguration that relieves TIR^RPS4^ domain inhibition by the TIR^RRS1^ domain. The TIR domain of sensor NLR RRS1-R lacks the NADase active site as well as the aspartate residue in the BB loop, which is also lost in other sensor NLRs of the paired TIR-NLR type in *Arabidopsis*. Our study reports a mechanism of effector-dependent *Arabidopsis* paired TIR-NLR activation that ensures rapid immune activation upon effector recognition.

## Results

**Fig. 1.**
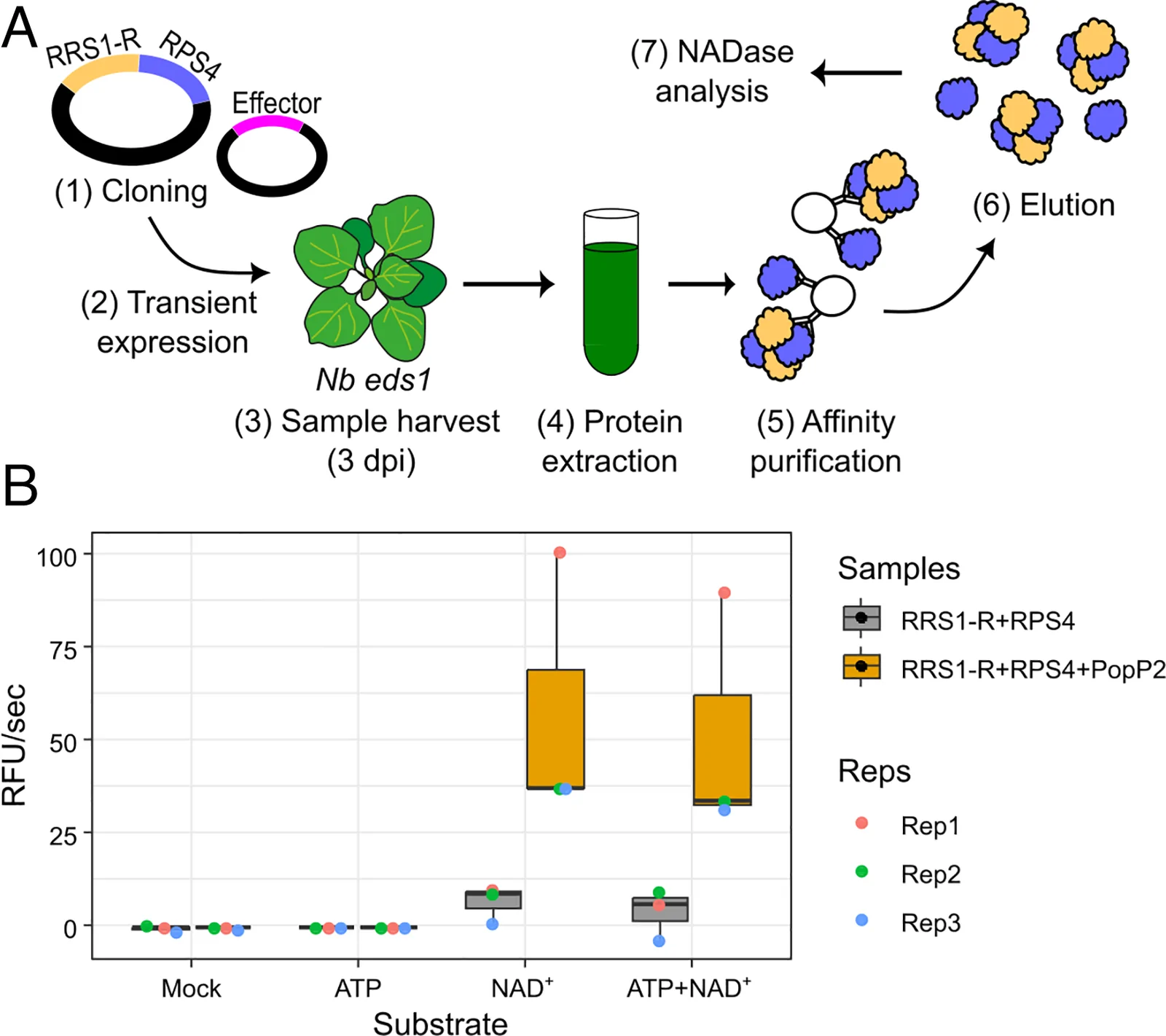
RRS1-R/RPS4 complex hydrolyzes NAD^+^ upon effector-dependent activation. (*A*) Schematic diagram of RRS1-R/RPS4 complex purification from transient expression in *N. benthamiana eds1* (*Nb eds1*). (*B*) NADase activity of purified RRS1-R/RPS4 complex coexpressed with or without effector PopP2. Purified RRS1-R/RPS4 complex from A were incubated with substrates ATP, εNAD^+^, or ATP+ εNAD^+^. Fluorescence emission by εNAD^+^ cleavage is measured in time-course of 1 min for 1 h, and reaction rate was plotted as Fluorescence/s.

We tested whether activated RRS1-R/RPS4 can hydrolyze NAD^+^ by purifying the complex in the presence or absence of effector PopP2. RRS1-R-twinStrep (RRS1-R fused with twinStrep tag) and RPS4-Flag (RPS4 fused with 3xFlag tag) were transiently expressed in *Nicotiana benthamiana eds1* mutant with or without effector and purified using anti-Flag agarose beads, followed by elution with 3xFlag peptide (Fig. 1*A* and *SI Appendix*, Fig. S1*A*). *N. benthamiana eds1* cannot activate cell death after RRS1-R/RPS4 coexpression with effector (29). εNAD^+^ is an analog of NAD^+^ that produces fluorescent εADP-ribose product upon cleavage by NAD^+^ hydrolases (30). We observed an increase in fluorescence resulting from hydrolysis of εNAD^+^ only from the active RRS1-R/RPS4 complexes coexpressed with cognate effector PopP2, and minimal changes in fluorescence from the inactive RRS1-R/RPS4 complex or without εNAD^+^ substrates (Fig. 1*B*). Adding ATP did not make any difference in the NAD^+^ cleavage, consistent with previous results using TIR^RPS4^ domain (31). The purified RRS1-R/RPS4 complex loaded on BN-PAGE migrated at the same position regardless of effector coexpression (*SI Appendix*, Fig. S1*B*).

We investigated the ratio between RRS1-R and RPS4 in the complex by tandem-affinity purification of RRS1-R and RPS4 (*SI Appendix*, Fig. S1*C*) transiently coexpressed from *N. benthamiana eds1* (*SI Appendix*, Fig. S1*D*) or wild-type (*SI Appendix*, Fig. S1*E*). Protein extracts were first immunoprecipitated with anti-Flag beads followed by elution with 3xFlag peptide and then immunoprecipitation with Streptactin beads (*SI Appendix*, Fig. S1*D*) or GFP beads (*SI Appendix*, Fig. S1*E*). The tandem-affinity purified samples were then visualized with Coomassie staining (*SI Appendix*, Fig. S1 *D* and *E*). Sequential affinity selection for each protein provided better information about the stoichiometry of the RRS1-R/RPS4 complex. Given all the tandem affinity purification experiments carried out with different tagged versions of RRS1-R and RPS4, we conclude that the most parsimonious interpretation of the stoichiometry of the RPS4/RRS1 complex is a 1:1 molar ratio (*SI Appendix*, Fig. S1 *D* and *E*).

To investigate native protein complex formation, we assessed protein migration of RRS1-R and RPS4 in BN-PAGE with or without coexpression. RRS1-R-Myc (RRS1-R fused with 4xMyc tag) and RPS4-Flag were extracted after transient coexpression in *N. benthamiana eds1* mutant leaves. Both proteins consistently migrated in BN-PAGE at a size between ~720 and ~1,020 kDa (*SI Appendix*, Fig. S2 *A*–*C*) upon coexpression. In the absence of its counterpart, both RPS4-Flag (*SI Appendix*, Fig. S2*A*) and RRS1-R-Myc (*SI Appendix*, Fig. S2*B*) migrated faster. We conclude that both RRS1-R and RPS4 associate with each other, consistent with previous reports (17, 32). Huh et al. (15) reported that RPS4 coimmunoprecipitates with itself only in the presence of RRS1 using a different protein extraction condition from a different genotype (wild-type instead of *eds1*) of *N. benthamiana* from that used here (15). However, in our conditions, both RRS1 and RPS4 alone show weak self-association (*SI Appendix*, Fig. S2 *A* and *B*), consistent with the auto-activity of RPS4 previously reported (15, 33). We also observed increased accumulation of both RRS1-R and RPS4 upon coexpression, suggesting they stabilize each other (*SI Appendix*, Fig. S2*C*).

**Fig. 2.**
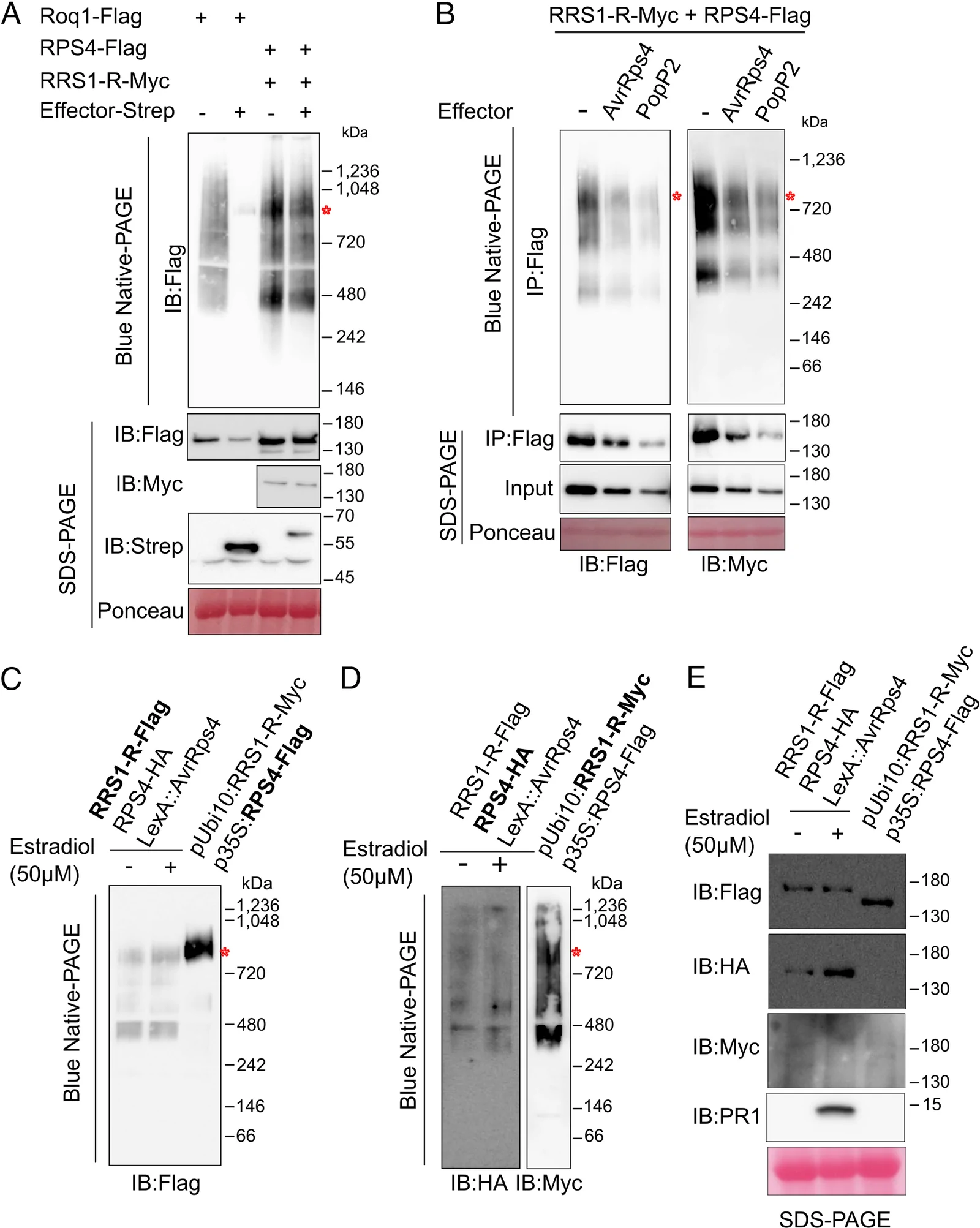
RRS1-R/RPS4 complex does not change in size before and after activation. (*A*) Complex formation of RRS1-R/RPS4 paired NLR and singleton TIR-NLR Roq1 upon activation by cognate effector (Strep-XopQ for Roq1, and PopP2-Strep for RRS1-R/RPS4). Proteins were extracted from *Nb eds1* expressing Roq1 and RRS1/RPS4 in the presence or absence of effector, loaded on BN-PAGE, followed by immunoblotting. Asterisk indicates RRS1-R/RPS4 complex. Aliquot of the samples were denatured, loaded on SDS-PAGE, followed by immunoblotting. Ponceau S staining serves as loading control. Molecular weights of markers are shown on the right. Similar results were obtained at least three times. (*B*) RRS1-R/RPS4 complex migration in the presence and absence of effector AvrRps4 and PopP2. Proteins were extracted from *Nb eds1* expressing RRS1-R/RPS4 in the presence or absence of effector AvrRps4 or PopP2, immunoprecipitated with anti-Flag beads, and eluted. These samples were loaded on BN-PAGE, followed by immunoblotting. Molecular weights of markers are shown on the right. Aliquot of samples were denatured, loaded on SDS-PAGE, followed by immunoblotting. Asterisk indicates RRS1-R/RPS4 complex. Ponceau S staining serves as loading control. Molecular weights of markers are shown on the right. Similar results were obtained at least three times. (*C* and *D*) *Arabidopsis* transgenic lines expressing RPS4-HA with RRS1-R-Flag with estradiol-inducible AvrRps4 or RPS4-Flag with RRS1-R-Myc, loaded on BN-PAGE, followed by immunoblotting. Asterisk indicates RRS1-R/RPS4 complex, and detected proteins in each panel are shown in bold. Molecular weights of markers are shown on the right. Similar results were obtained at least three times. (*E*) Samples from *C* and *D* were denatured, loaded on SDS-PAGE, followed by immunoblotting. PR1 expression was checked with anti-PR1 antibody. Ponceau S staining serves as loading control. Molecular weights of markers are shown on the right.

Interestingly, unlike the oligomerization of Roq1 induced upon effector-dependent activation (34), no additional migration retardation was observed in the RRS1-R/RPS4 complex in the presence of PopP2 (Fig. 2*A*). To confirm the comigrating bands of RRS1-R and RPS4 are indeed a complex, we immunoprecipitated RPS4-Flag and loaded on BN-PAGE and visualized both RPS4-Flag and RRS1-R-Myc (Fig. 2*B*). We observed comigration of both RRS1-R and RPS4 after immunoprecipitation, which became more prominent upon immunoprecipitation compared to lysate conditions (*SI Appendix*, Fig. S2*D*). Coexpression with cognate effectors AvrRps4 or PopP2 had no effect on oligomeric state (Fig. 2 *A* and *B* and *SI Appendix*, Fig. S1*B*).

To test if RRS1-R/RPS4 properties in *Arabidopsis* resemble those in *N. benthamiana*, we performed BN-PAGE on protein extracted from *Arabidopsis* transgenic lines (Fig. 2 *C*–*E*). One line expressed RPS4-HA and RRS1-R-Flag under moderate expression promoters with estradiol-inducible AvrRps4-expression (32), and another *Arabidopsis* transgenic line overexpressed RPS4-Flag and RRS1-R-Myc, driven by 35S promoter and Ubi10 promoter, respectively (*SI Appendix*, Fig. S2 *E* and *F*). Both RRS1-R-Flag and RPS4-Flag comigrated in BN-PAGE (Fig. 2*C*), consistent with our results using transient expression in *N. benthamiana eds1*. RRS1-R and RPS4 thus form a complex in *N. benthamiana* indistinguishable from that formed in *Arabidopsis* (Fig. 2*E*). Estradiol-mediated induction of AvrRps4 did not alter the migration of the RRS1-R/RPS4 complex in BN-PAGE (Fig. 2*C*), despite induction of PR1 expression which suggests activation of defense (Fig. 2*E*).

Previously, the crystal structure of TIR^RRS1^ and TIR^RPS4^ domains and their heterodimer revealed their structure, each comprising a five-stranded parallel β sheet (βA to βE) and five α-helical regions (αA to αE) positioned in tandem, with the heterodimeric interface formed between αA and αE, designated the AE interface (17) (*SI Appendix*, Fig. S3*A*). This AE interface is also required to configure NADase activity in the active TIR^Roq1^ tetramer compared to inactive TIR^RUN1^ monomer (35) (*SI Appendix*, Fig. S3*B*), while this AE interface-mediated interaction of TIR^RRS1^ and TIR^RPS4^ is crucial for repression of RPS4 TIR domains (17). The AE interface in TIR^Roq1^ enables the formation of the BE interface-dependent dimer of dimer conformation. It is specifically the BE interface that becomes the NADase active site as well as NAD^+^ binding pocket. A conserved glutamate (E) residue in the αC of the TIR domain (5, 6), and the BB-loop connecting αB and βB at the BE interface is required for NADase activity (8, 9) (*SI Appendix*, Fig. S3*A*). Only one TIR protomer from the dimer through AE interface provides the NADase active site, and thus singleton TIR^Roq1^ oligomerizes to form two NADase active sites, from the two BE interfaces in the tetramer (8, 9).

**Fig. 3.**
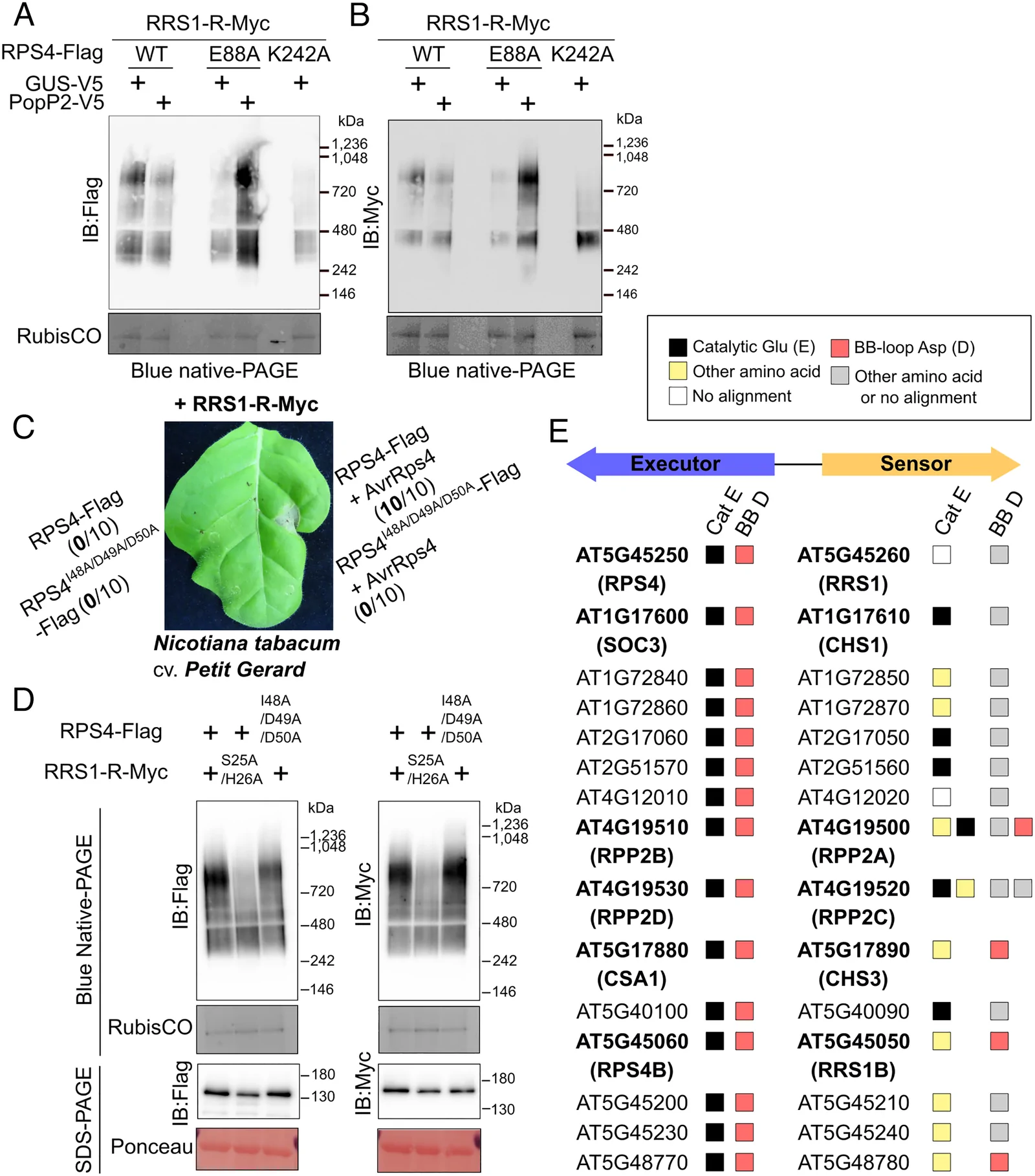
AE interface of RRS1-R TIR domain is required for RRS1/RPS4 hetero-oligomer formation, but NADase activity is not required for hetero-oligomerization. (*A* and *B*) RRS1-R/RPS4 complex upon coexpression of effector and loss of NADase activity. Proteins were extracted from *Nb eds1* plants expressing RPS4 wild-type or E88A mutant, with RRS1-R and in the presence or absence of effector PopP2. RPS4 P-loop mutant (RPS4^K242A^) without effector is also expressed with RRS1-R-Myc. Protein lysates were loaded on blue native-PAGE, followed by immunoblotting with Flag (*A*) or Myc (*B*) antibody. Similar results were observed at three times, of which representative image is shown. Molecular weights of markers are shown on the right. (*C*) *Nicotiana tabacum* cv. *Petit Gerard* leaves expressing RRS1-R with RPS4 wild-type or RPS4 BB-loop mutation (RPS4^I48A/D49A/D50A^-Flag) in the presence or absence of effector AvrRps4 were tested for HR. Images were taken 5 dpi, and at least three leaves were used for each replicate. Number of HR occurrences (in bold) out of total number of leaves tested are indicated in parentheses. (*D*) Proteins from *Nb eds1* leaves expressing RRS1-R/RPS4 complex with either AE mutation (RRS1-R^S25A/H26A^-Myc) or BB-loop mutation (RPS4^I48A/D49A/D50A^-Flag) was extracted, loaded on BN-PAGE or SDS-PAGE (after denaturation), followed by immunoblotting. RubisCO complex, and Ponceau S staining serves as loading control. Molecular weights of markers are shown on the right. (*E*) Presence or absence/mutation of catalytic glutamate (Cat E) and aspartate residue in BB-loop (BB D) in paired TIR-NLRs. Known and predicted paired TIR-NLRs were selected, and amino acids in the corresponding structural positions to the Roq1 catalytic glutamic acid (E86), conserved BB-loop aspartic acid (D45) were extracted from alignments and shown. Data based on *SI Appendix*, Fig. S5.

We tested whether these motifs in the TIR domains are required for oligomerization and/or their effector recognition function. TIR^RPS4^ contains both the NADase active site and the BB loop (*SI Appendix*, Fig. S3*A*). We observed that mutation of RPS4 E88 into alanine (A) did not abrogate oligomerization of RRS1-R/RPS4 complex, regardless of effector coexpression (Fig. 3 *A* and *B*). In contrast, the NB-ARC P-loop mutation (K242A) of RPS4 led to loss of oligomerization (Fig. 3 *A* and *B*). Both the E88A mutant and the K242A mutant of RPS4 led to loss of HR (*SI Appendix*, Fig. S3 *C* and *D*).

After transient expression with RPS4-Flag in *N. benthamiana eds1,* the RRS1-R AE interface mutant (RRS1-R^S25A/H26A^-Myc; alanine substitution of both S25 and H26) shows significantly reduced RRS1-R/RPS4 complex abundance in BN-PAGE (Fig. 3*D* and *SI Appendix*, Fig. S3*E*). In this *eds1* background, RPS4-Flag failed to coimmunoprecipitate RRS1-R^S25A/H26A^ -Myc (*SI Appendix*, Fig. S3*F*). This indicates that the AE interface, originally identified as the binding interface of the TIR domains, plays an important role in the dimerization as well as the higher-order oligomerization of the full-length RRS1-R/RPS4 complex.

We observed that, similar to the NADase active site mutation, the BB-loop mutation of RPS4 (RPS4 ^I48A/D49A/D50A^; alanine substitutions of I48, D49, and D50) did not result in reduced oligomerization (Fig. 3*D*), while HR was not observed when RPS4 ^I48A/D49A/D50A^ was coexpressed with RRS1-R and effector AvrRps4 (Fig. 3*C*). TIR^RRS1-R^ lacks NADase active site, and has a shorter BB loop, which likely abolishes NADase activity (8) (*SI Appendix*, Fig. S3*A*). The TIR domains of the sensor NLR in other TIR-NLR pairs also lack the NADase active site, similar to RRS1-R (6). To investigate whether BB-loop sensor mutations are common in paired NLRs in *Arabidopsis*, we identified 152 unique TIR domains from the *Arabidopsis* Col-0 proteome, and constructed a phylogenetic tree. The structure of each TIR domain was predicted using AlphaFold2 (36), and then aligned with TIR^Roq1^. The residue that aligns with the catalytic glutamate residue of TIR^Roq1^, and the residue that aligns with aspartate residue in the BB-loop of TIR^Roq1^, were visualized (*SI Appendix*, Fig. S4).

In most TIR domains, a conserved glutamate residue was identified at the catalytic site (Cat E), and conserved aspartate residue was identified in the BB-loop region (BB-D, third residue within BB-loop) (*SI Appendix*, Fig. S4). However, we observed that some TIR domains lack the catalytic glutamate residue or aspartate residues from the BB-loop (*SI Appendix*, Fig. S4). This clade includes many of the sensor NLRs from paired TIR-NLRs found in *Arabidopsis* Col-0 (Dataset S1*A*) (37). In all cases, one TIR domain, belonging to the sensor, lacked the catalytic glutamate residue or the aspartate residue in the BB-loop (Fig. 3*E*) (38). In contrast, executor NLRs of the paired TIR-NLRs retain the catalytic glutamate residue and the aspartate residue in the BB-loop predicted to be required for NADase activity (Fig. 3*E*). This suggests the sensor TIR domains may lack NADase activity, consistent with a recent study of NADase activity of TIR domain proteins in *Arabidopsis* (38).

Next, we tested whether the NB-ARC domain is required for oligomerization. Mutation of the P-loop motif of RPS4 (K242A) abrogates effector-dependent HR, whereas the RRS1-R P-loop mutant (K185A) did not impact effector-dependent HR (17). We observed slow-migrating forms of RRS1-R/RPS4 were lost in RPS4^K242A^ similar to RRS1-R^S25A/H26A^ and accumulation was reduced with RRS1-R^K185A^ (*SI Appendix*, Figs. S3 *A* and *B* and S5*A*). Some NB-ARC mutations lead to autoactivation, and HR caused by these mutations in RPS4 was enhanced by RRS1-R overexpression (33). We tested RRS1-R/RPS4 complex formation by the S270F mutation of RPS4 NB-ARC domain, and *slh1* mutation of RRS1-R (39). We observed that both autoactive mutations assembled complexes indistinguishable on blue native-PAGE from the inactive RRS1-R/RPS4 complex, as well as the effector-activated RRS1-R/RPS4 (*SI Appendix*, Fig. S5*B*). Thus, we conclude that while P-loop mutations of RPS4 abolish oligomerization, autoactive mutations of RRS1 or RPS4 do not alter migration of the RRS1-R/RPS4 complex.

We noticed that the RRS1-R/RPS4 complex dissociates at high concentrations of DTT (10 mM), only upon coexpression of RPS4 and RRS1-R (*SI Appendix*, Fig. S6*A*). This prompted us to test whether disulfide bonds form within the RRS1-R/RPS4 complex, either as intramolecular bonds, or as intermolecular bonds between RRS1-R and RPS4. Conceivably, the slower-migrating form of RPS4 and RRS1-R results from oxidation upon extraction (*SI Appendix*, Fig. S6*B*). To prevent oxidation of protein extracts generating artifactual disulfide bonds during extraction, we added N-ethylmaleimide (NEM) to the protein extract to prevent free sulfhydryl groups from forming disulfide bonds in vitro. After purification of proteins from *N. benthamiana*, multiple disulfide bonds were identified through LC–MS/MS (*SI Appendix*, Figs. S6*C*, S7, and S8). We used two different disulfide linkage analysis software, MASCOT and DBond (40). DBond analysis retrieved fewer disulfide bonds, although the top 6 disulfide linkages were highly similar between the two analyses (*SI Appendix*, Fig. S6*C* and Dataset S1 *B* and *C*). We measured the distance between the identified cysteine residues from the AlphaFold3 prediction model of RPS4 and RRS1-R (*SI Appendix*, Fig. S6 *D* and *E*). Except for RPS4 C594-C598 RRS1-R C289-C291, and RRS1-R C1233-C1241 disulfide bonds, other bonds showed secondary structures that are likely to prevent disulfide formation (*SI Appendix*, Fig. S6 *D* and *E*).

We chose three predicted disulfide linkages, RPS4^C783^-RPS4^C1020^, RPS4^C1001^-RPS4^C1006^, and RRS1-R^C289^-RRS1-R^C291^ and tested alanine substitutions of these residues for effector recognition (*SI Appendix*, Fig. S9*A*). Both RRS1-R C289 and C291 to alanine mutation, and RPS4 C1001 and C1006 to alanine mutation retain effector-dependent HR. However, we observed that mutation of RPS4 C783 to alanine abrogated effector-dependent HR (*SI Appendix*, Fig. S9*A*). To preserve the polarity of the cysteine group, we mutated RPS4 C783 to serine and also mutated RPS4 C1020 to serine which was predicted to form a disulfide linkage with C783 (*SI Appendix*, Fig. S6 *C* and *D*). However, only C783S showed loss of HR, whereas C1020S resembles wild-type (*SI Appendix*, Fig. S9*B*).

**Fig. 4.**
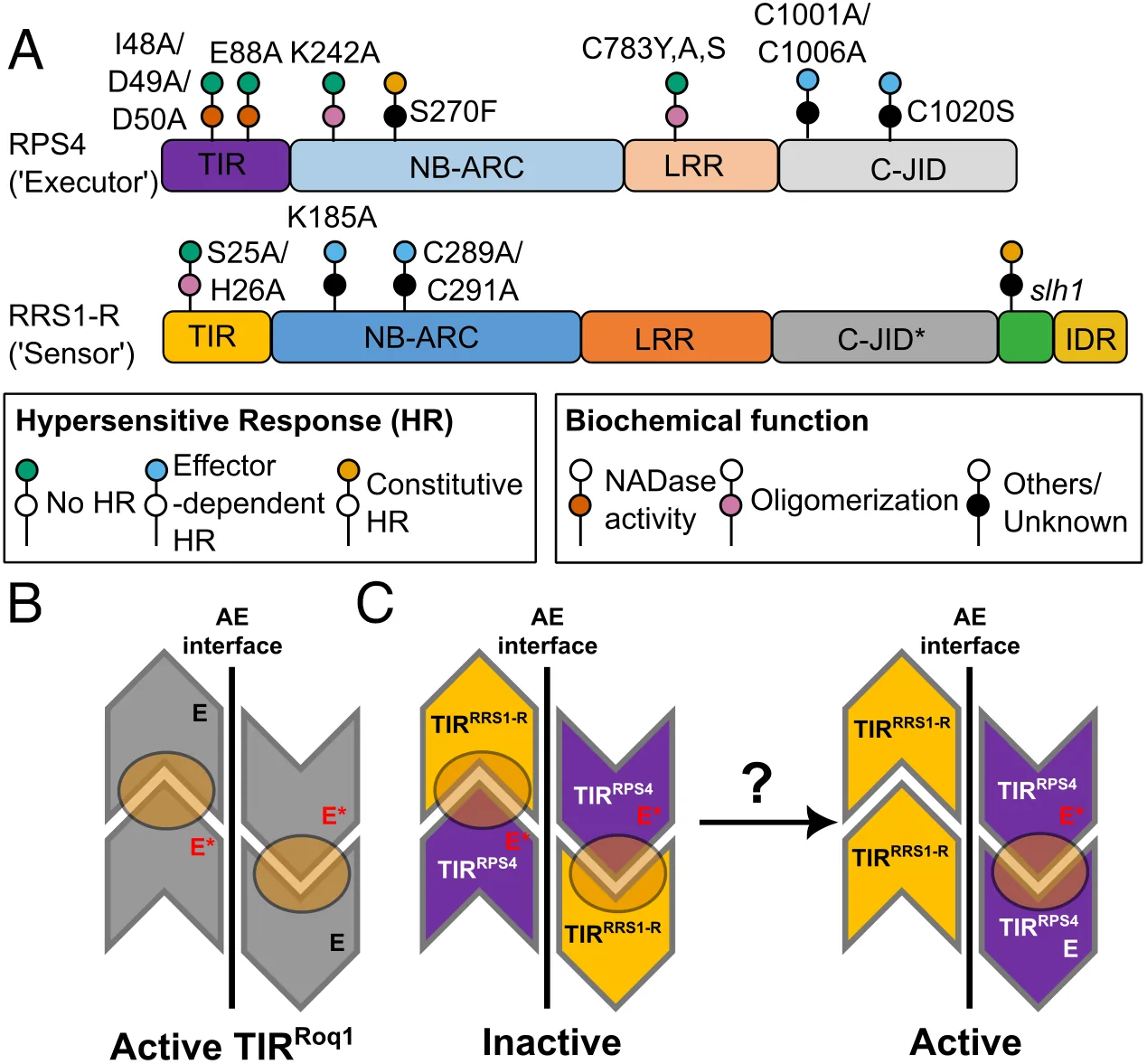
Summary of RRS1-R/RPS4 complex formation and model of possible TIR conformations. (*A*) Summary of HR phenotype (green, no HR; blue, effector-dependent HR; yellow, autoactive HR) and biochemical function (orange, NADase enzyme activity; pink, oligomerization; black, other/unknown function) of the mutant residues tested in this study. (*B*) Schematic diagram of TIR^Roq1^ homotetramer with two NADase active sites (yellow circle). Catalytic glutamate residue is shown as E*. (*C*) Schematic diagram of possible arrangement of TIR^RRS1^ and TIR^RPS4^ heterotetramer before and after activation.

Previous suppressor screens of autoactive mutant of RRS1-R identified multiple residues in RPS4 required to suppress the autoactive *slh1* allele of RRS1-R (41). One of the mutants identified (*sushi86*) carried C783Y mutation of RPS4. Seedlings of RPS4 C783Y mutation grew similar to No-0, despite having the *slh1* background (*SI Appendix*, Fig. S9*C*). When RPS4 C783S was transiently expressed in *N. benthamiana eds1* and the protein extracts loaded on BN-PAGE, oligomers of RRS1-R/RPS4 were lost (*SI Appendix*, Fig. S9*D*). We also noticed reduction of RPS4^C783S^-Flag (*SI Appendix*, Fig. S9*E*), suggesting RPS4 C783 may be involved in structural stabilization of the RRS1-R/RPS4 complex or in recognition of the activated state of RRS1-R. Indeed, this C783 is conserved across other TIR-NLRs, and is part of the LRR motif (LxxLxxLxxLxLxx(**N/C/T**)x(x)LxxIPx) (42). Other predicted disulfide linkages, C289-C291 and C1001-C1006 had no effect in RRS1-R/RPS4 complex formation (*SI Appendix*, Fig. S9 *F* and *G*) or effector-dependent activation of HR (*SI Appendix*, Fig. S9*A*). We conclude that multiple domains are required to maintain RRS1-R/RPS4 complex oligomerization and function. While enzymatic activity alone is not required for oligomerization, it is required for eliciting HR upon effector recognition (Fig. 4*A*).

We next sought to identify conformational changes that may correlate with activation of the RRS1-R/RPS4 complex, without altering its stoichiometry. For this, we employed AlphaFold3 multimer (43) to test possible assembly of the TIR^RPS4^ and TIR^RRS1^ domains (*SI Appendix*, Fig. S10). Upon activation, the Roq1 homotetramer forms a dimer of a dimer conformation, assembling two NADase active sites (Fig. 4*B*). We observed that TIR^RPS4^ or TIR^RRS1^ domains alone could not assemble into the Roq1 homotetramer conformation (*SI Appendix*, Fig. S10). However, upon inclusion of NAD^+^, TIR^RPS4^ predominantly assembled into Roq1-like homotetramer, which was not observed for TIR^RRS1^ (*SI Appendix*, Fig. S10). When 2 molecules of TIR^RPS4^ and TIR^RRS1^ domains were modeled together, 4/5 predicted models showed a dimer of a heterodimer assembly, with a heteromeric BE interface (*SI Appendix*, Fig. S10 and Fig. 4*C*). Interestingly, upon addition of NAD^+^ to the heterotetrameric TIR^RPS4^ and TIR^RRS1^ model, the two best predicted models formed a homomeric BE interface, resulting in one BE interface comprised of two TIR^RPS4^ domains within the heterotetramer (*SI Appendix*, Fig. S10 and Fig. 4*C*).

## Discussion

Paired NLRs are found in diverse plant species and play important roles in disease resistance in crops (13). Transfer of paired NLRs together between species can elevate resistance to disease (29, 44). Such paired NLRs can also provide a chassis to create novel disease resistance by substituting one integrated domain for another integrated domain (45–50). Despite the remarkable progress in structural analysis of activated NLRs, we still have much to learn about paired NLR immune receptors. The diversity of paired NLRs, and the distinction between paired TIR-NLRs and paired CC-NLRs, suggest the potential for mechanistic differences between paired NLRs.

Which interfaces of each NLR are involved in complex formation? We observed that mutations in each of the TIR (Fig. 3 *A, B,* and *D*), NB-ARC (Fig. 3 *A* and *B* and *SI Appendix*, Fig. S5*A*), and LRR (*SI Appendix*, Fig. S9) domains of RRS1-R and/or RPS4 can compromise formation of an RRS1-R/RPS4 complex. This is in contrast to a previous report using different constructs suggesting full-length RRS1-R and RPS4 interact even with TIR domain AE interface mutations (17). However, using new constructs with different tags, and different protein extraction conditions, we now find that RRS1-R-Myc AE interface mutants cannot be coimmunoprecipitated by RPS4-Flag wild-type proteins (*SI Appendix*, Fig. S3*F*). This is consistent with the results obtained from another paired TIR-NLR CHS3/CSA1 (18), and suggests TIR^RPS4^-TIR^RRS1^ interactions are essential for complex formation.

We show here that there are no changes in size of the RRS1-R/RPS4 complex in the presence or absence of cognate effector (Fig. 2). This is consistent with previous studies on RRS1-R and RPS4 interactions that show no apparent change upon coexpression with a cognate effector (15, 32) while displaying changes in proximity of TIR^RPS4^ and TIR^RRS1^ upon effector recognition (28). This constitutive interaction between RRS1-R and RPS4 may have selected for loss of NADase activity in the RRS1-R sensor, reducing the risk of constitutive NADase activity. The sensor NLRs of most paired TIR-NLRs have degenerate BB loops and lack the NADase active site (Fig. 3*E*), as well as degenerate C-JID (51), in addition to their genetic linkage to an executor TIR-NLR and the presence of ID. This feature may help identify novel paired TIR-NLRs in different plant genomes, especially those that have sensor NLRs without an ID (52).

Homomeric TIR-domain protein SARM1 is also oligomeric before activation (53). SARM1 assembles into an octameric ring in the inactive state (54), which in turn becomes a two-strand, antiparallel octamer (“tetramer of a dimer”) upon provision of NAD^+^ precursor NMN (Nicotinamide mononucleotide) (53). A similar mechanism of conformational change is likely to occur with the paired NLR RRS1-R/RPS4 complex. In conclusion, our analyses suggest that the *Arabidopsis* RRS1-R/RPS4 paired NLR complex is in a “poised” state as a dimer of a heterodimer prior to effector recognition. Another report regarding the *Arabidopsis* paired NLR CHS3/CSA1 showed oligomerization into a heterotetramer (18) but was not able to address recognition-dependent changes. We present here a mechanism whereby heteromeric NLRs can rapidly respond to ligand perception without altering stoichiometry or forming new protein complexes. This may make defense activation more efficient since unlike activation of singleton TIR-NLRs, it avoids the need for effector-engaged, “activated” monomers to associate with three additional activated monomers to form a tetramer.

## Materials and Methods

Detailed description of materials and methods in this study can be found in *SI Appendix*.

### Plant Materials.

*N. benthamiana* wild-type and *eds1* plants (55), and *N. tabacum* cv. Petit Gerard plants were grown in long-day conditions (16 h light/8 h dark) at 22 °C, humidity 45 to 65%. *Arabidopsis* transgenic lines (Dataset S1*D*) were grown in short-day conditions (8 h light/16 h dark) at 22 °C for 4 to 5 wk, then moved to long-day conditions for seed propagation. Transgenic *Arabidopsis* line seedlings were grown in 1/2 MS liquid culture with 1% sucrose for 7 d under constant light at 22 °C.

### Golden Gate Cloning.

To generate constructs of epitope tag-fused RRS1-R or RPS4, Golden Gate cloning (56) was used. Generated constructs are listed in Dataset S1*E*. All the modules were provided by TSL Synbio team.

### Agrobacterium Infiltration.

Binary constructs for transient expression were transformed into *Agrobacterium tumefaciens* strain GV3101-pMP90. *Agrobacterium* cells were resuspended in infiltration buffer (10 mM MgCl_2_ and 10 mM MES, pH 5.6, 100 μM acetosyringone). OD600 of each construct-containing *Agrobacterium* was adjusted to 0.5, and OD600 for *Agrobacterium* containing p19 was adjusted to 0.2. Infiltration was performed after 1 to 2 h incubation at room temperature. The infiltrated *N. benthamiana* leaves were harvested 3 d post infiltration (3 dpi).

### HR Assay.

Agrobacterium cells harboring the constructs were resuspended in infiltration buffer and subsequently infiltrated into 4- to 5-wk-old *N. tabacum* cv. Petit Gerard plants. The leaves were imaged at 5 dpi.

### Protein Extraction for Blue-Native PAGE Analysis.

Protein extraction was performed as described in Ahn et al. (57) with minor adjustments. Agrobacterium-infiltrated *N. benthamiana* leaves or *Arabidopsis* seedlings/rosette leaves were harvested, and immediately frozen in liquid nitrogen. Frozen samples were then ground with Geno/Grinder® (SPEX SamplePrep). Protein extraction buffer (50 mM Tris-Cl pH 7.5, 150 mM NaCl, 10% Glycerol, 5 mM MgCl_2_, 1 mM DTT, 5 mM ascorbic acid, 0.2% NP-40, protease inhibitor cocktail) was added to the ground tissue and vortexed. Centrifugation was performed at 18,000×*g* for 15 and 5 min subsequently to remove cell debris. Sample aliquots were either directly used for blue native-PAGE analysis, or immediately frozen in liquid nitrogen. Samples for SDS-PAGE were denatured with 3× SDS sample buffer (stock concentration 30% glycerol, 3% SDS, 93.75 mM Tris-Cl pH 6.8, 0.06% bromophenol blue) with 100 mM DTT at 70 °C for 10 min. Additional detail for large-scale immunoprecipitation can be found in *SI Appendix*.

### Blue Native-PAGE.

Protein extracts and immunoprecipitation (IP) eluates were added with 4× NativePAGE^TM^ Sample Buffer (Invitrogen^TM^, BN2003) and 1% Coomassie G-250 to a final concentration of 0.125%. Blue native-PAGE was performed with these samples as described in Ahn et al. (58). NativeMark^TM^ Unstained Protein Standard (Invitrogen^TM^, LC0725) was used as molecular weight markers. Further information regarding immunoblotting can be found in *SI Appendix*.

### Phylogenetic Tree Analysis of *Arabidopsis* TIR Domains.

~152 proteins with homology to TIR domains were selected from the *Arabidopsis* Col-0 proteome (Araport11_pep_20220914) for analysis. Detailed information of sequence alignment, protein structure prediction, and results visualization can be found in *SI Appendix*.

### Sample Preparation for Mass Spectrometry.

To identify the disulfide bonds in RRS1-R/RPS4 complex, 4- to 5-wk-old *N. benthamiana* plants were used for infiltrating 3xFlag-tagged RPS4 and 4xMyc-tagged RRS1-R, sampled 3 dpi. Protein extraction was carried out with the following buffer: 50 mM HEPES pH 7.0, 150 mM NaCl, 10% Glycerol, 5 mM MgCl_2_, 10 mM NEM (N-ethylmaleimide), 0.2% NP-40, 0.2 mM PMSF, protease inhibitor cocktail, 2% PVPP. RPS4 were enriched using anti-Flag M2 beads (A2220, Merck), and eluted using 3xFlag peptide (F4799, Merck). Samples were incubated with SDS only and fractionated using SDS-PAGE. Gels that have corresponding size to RPS4 and RRS1-R were excised and in-gel digested by trypsin. Extracted peptides were measured for mass spectrometry analysis. Further information regarding LC–MS/MS analysis, and disulfide bond identification can be found in *SI Appendix*.

### NADase Activity Assay.

Fluorescence emitted from εNAD^+^ catalysis was measured to examine the NADase activity of the purified RRS1-R/RPS4 complex (59). Briefly, RRS1-R/RPS4 complex was purified with anti-Flag M2 beads from transient expression in *N. benthamiana eds1* plants and eluted with 3xFlag peptide. 15 µL eluates were mixed with 100 μM εNAD^+^ (Merck, N2630) with or without 1 mM ATP. Reactions were measured using Spectra ID5 Max at 1-min intervals for 1 h, excitation 310 nm and emission 400 nm. Three technical replicates were measured for each biological replication.

## Supplementary Material

Appendix 01 (PDF)

Dataset S01 (XLSX)

## Data Availability

Mass spectrometry dataset data have been deposited in PRIDE (https://doi.org/10.6019/PXD062781) (60).
