# Supplementary material for: Recognition-dependent activation of the Arabidopsis RRS1-R/RPS4 TIR-NLR immune receptor pair
Source: Proc Natl Acad Sci U S A. 2026 Oct 27;123(44):e2611802123. doi: 10.1073/pnas.2611802123 (PMC13732033; doi:10.1073/pnas.2611802123)
Supplement: Supplementary file 1 [file pnas.2611802123.sapp.pdf]

PNAS

Supporting Information for
Recognition-dependent activation of the Arabidopsis RRS1-R/RPS4 TIR-NLR immune receptor pair

Hee-Kyung Ahn[1,2,*], Guanghao Guo[2,3], Jan Skłenar[2], Shania Pin Yin Keh[2], Sung Un Huh[2,4], Michelle T. Hulin[2,5], Hayden Burdett[1], Maria Sindalovskaya[2,6], Seungjin Na[7,8], Jihyeon Choi[2], Nitika Mukhi[6], He Zhao[2], Lena S. Knorr[2], Eunok Paek[9], Mark J. Banfield[6], Frank L.H. Menke[2], Wenbo Ma[2], Jonathan D.G. Jones[2,*]

Correspondence: Jonathan D.G. Jones (jonathan.jones@tsl.ac.uk), Hee-Kyung Ahn (hahn2@ed.ac.uk)

This PDF file includes:

Supporting text
Figures S1 to S10
SI References

Other supporting materials for this manuscript include the following:

Dataset S1

Supporting Methods

Protein extraction for large-scale immunoprecipitation

18 *N. benthamiana eds1* plants were infiltrated with Agrobacterium for transient expression. 2-3 leaves from each plant were infiltrated in 4-week old *N. benthamiana* plants. Leaves were harvested at 3 dpi, resulting in fresh weight of 15-20 g. These samples were immediately frozen in liquid nitrogen, and then ground with mortar and pestle, while maintaining the apparatus cold. Protein extraction buffer (50 mM HEPES pH 7.0, 150 mM NaCl, 10% Glycerol, 5 mM $MgCl_2$, 1 mM DTT, 5 mM ascorbic acid, 0.2% NP-40, 0.2 mM PMSF, protease inhibitor cocktail, 2% PVPP) was added in 1 (tissue): 5 (buffer) ratio. Insoluble PVPP and cell debris were separated with centrifugation of 4,500 g for 15 min. After filtering the lysate with nylon mesh, the supernatants were centrifuged once more with 45,000 g for 45 min. The resulting supernatant was incubated with Flag M2 agarose beads (Merck A2220) for 1 h, and washed with extraction buffer without PVPP. Samples were eluted with 3xFlag peptides (Merck F4799) for 1 h.

Immunoblotting

Protein samples incubated with SDS sample buffer were loaded on SDS–PAGE gels and run at 90 V. After dye front reached the end, these gels were transferred with Trans-Blot® Turbo™ Transfer System (Biorad, #1704150) at conditions of 1.0 mA for 30 min onto PVDF membranes. Transferred membranes were blocked with 5% skimmed milk in TBS-T, and antibodies were added subsequently and incubated overnight at 4°C. The following antibodies were used; anti-Flag (Conjugated HRP, Merck, A8592), anti-Myc (Conjugated HRP, Merck, 16-213), anti-HA (Conjugated HRP, Roche, 12013819001), anti-StrepII (Merck, 71591-M), anti-Histone H3 (Agrisera, AS10 710), anti-PR1 (Agrisera, AS10 687), anti-Rabbit secondary (Conjugated HRP, Merck, AP132P). HRP Signals were detected using ECL substrates (Thermo Fisher, 34580). After detection, membranes were stained with Ponceau S solution (Merck, P7170) to use as loading control. PageRuler™ Prestained Protein Ladder (Thermo Scientific, 26616, size range 10-180kDa) or Spectra™ Multicolor High Range Protein Ladder (Thermo Scientific, 26625, size range 40-300kDa) was used as molecular weight markers.

Phylogenetic tree analysis of Arabidopsis TIR domains

The Arabidopsis Col-0 proteome (Araport11_pep_20220914) was downloaded from https://www.arabidopsis.org/. Interproscan v5(1) was ran on each protein. Python and unix scripting was then used to select 140 proteins with hits to TIR domains (using terms "TIR" and "domain"). Proteins with more than one TIR domain in different regions were split based on having non-overlapping hits. TIRs for each proteins slice variants were manually inspected and only those non-overlapping sequences and unique sequences were kept. Highly truncated sequences were also removed. This left 152 unique TIR domains. ClustalW 2.1(2) was used to create a 357 aa alignment. The alignment was trimmed of poorly aligned regions with trimAL v1.5(3) to 108 aa. IQ-

TREE v.2.3.3(4) utilized to create a maximum-likelihood phylogenetic tree using model JTT+F+G4 with 1000 bootstraps. The structure of each TIR domain was predicted with AlphaFold2(5). Alignments with the TIR domain from Roq1 (7jlx: S11-F176) were performed using frtmalign(6). Amino acids in the corresponding structural positions to the Roq1 catalytic glutamic acid (E86), conserved BB-loop aspartic acid (D45), the BB-loop (F43-K53) and surrounding region (E37-E58) were extracted from alignments using unix scripting. These results were visualized using R studio with packages ape, ggplot2, ggtree, ggmsa and ggnewscale(7-11). The tree was rooted to divergent TIR-N proteins AT5G56220.1 and AT4G23440.1. Code for these analysis are available at https://github.com/michhulin/Pseudomonas/tree/main/tirs.

LC-MS/MS Analysis

Extracted peptides were measured by Orbitrap Fusion in data-dependent acquisition method. The MS/MS fragmentation used both CID and HCD in parallel with each precursor ion selected for fragmentation. Raw files were peak-picked by MSConvert (Proteowizard project), searched by Mascot (Matrix Science) with added settings for disulfide crosslinks. The data could not be filtered to 1%FDR as current version of Mascot v2.8 does not support this validation. Mascot v2.8 does not support FDR validation for crosslinked search results, therefore a significance threshold $p < 0.05$ was used instead. The mass spectrometry proteomics data have been deposited to the ProteomeXchange Consortium via the PRIDE(12) partner repository with the dataset identifier PXD062781 and 10.6019/PXD062781.

D-Bond analysis

To confidently identify disulfide-linked peptides, a multi-stage search strategy was employed. First, a conventional database search was performed to identify linear peptides and contaminant peptides. MS/MS spectra that remained unidentified after this initial search were then subjected to a disulfide-linked peptide search. Finally, candidate disulfide-linked peptide identifications were evaluated by comparison with the results of an open modification search.

The initial search was performed using MSFragger (v4.2) through FragPipe (v23.0) (13). Search parameters were as follows: trypsin specificity, two enzymatic termini, up to two missed cleavages, precursor mass tolerance of ±20 ppm with ^{13}C isotope errors of 0 or 1, and fragment mass tolerance of ±0.4 Da. Oxidation of methionine, N-ethylmaleimide (NEM) of cysteine, and protein N-terminal acetylation were specified as variable modifications. The database consisted of the RRS1-R and RPS4 protein sequences together with protein sequences from *N. benthamiana*. Search results were filtered to 1% FDR using a target-decoy strategy (14), and MS/MS spectra that remained unidentified after this filtering step were subjected to the second-stage search.

The second-stage search for disulfide-linked peptides was performed using DBond (v3.02) (15). Search parameters were as follows: trypsin specificity, two enzymatic termini, up to two missed cleavages, precursor mass tolerance of ±10 ppm with ^{13}C isotope errors of 0, 1, 2, or 3, and

fragment mass tolerance of ±0.4 Da. Oxidation of methionine was allowed as a variable modification, and N-ethylmaleimide (NEM) was specified as the free-cysteine blocking reagent. The search database consisted of the RRS1-R and RPS4 proteins, and both inter-protein disulfide bonds between RRS1-R and RPS4 and intra-protein disulfide bonds were allowed.

Given that disulfide-linked peptide searches involve a substantially larger search space, they are more prone to false-positive identifications. To further verify DBond-derived candidates, spectra assigned to disulfide-linked peptides by DBond were re-evaluated to assess whether they could be better explained as modified linear peptides. To this end, an open modification search was performed using MODplus (v2.1) (16). Search parameters were as follows: precursor mass tolerance of ±10 ppm with ^{13}C isotope errors of 0, 1, 2, or 3; fragment mass tolerance of ±0.4 Da; and trypsin specificity with at least one enzymatic terminus. Missed cleavages were not restricted. All Unimod-listed modifications (17), including amino acid substitutions, were considered within a precursor mass shift range of –150 to +350 Da.

Finally, DBond assignments were manually verified against the corresponding MODplus-derived modified peptide assignments for each spectrum. A disulfide-linked peptide was accepted only when the DBond assignment explained the observed spectrum more convincingly than the corresponding MODplus assignment, based on fragment-ion matching patterns and overall spectral interpretability.

Structural modelling

Structural models of the TIR domains were generated using Google Deepmind's AlphaFold 3 server (18), using TIR domain sequences of RPS4 and RRS1-R. pTM and ipTM values were also taken from the AlphaFold 3 server. Structure alignments to determine if tetramers formed a ROQ1-like interface (PDB ID: 7JLX) and visualisation was done using UCSF ChimeraX (19). UCSF ChimeraX is developed by the Resource for Biocomputing, Visualization, and Informatics at the University of California, San Francisco, with support from National Institutes of Health R01-GM129325 and the Office of Cyber Infrastructure and Computational Biology, National Institute of Allergy and Infectious Diseases.

Figure S1

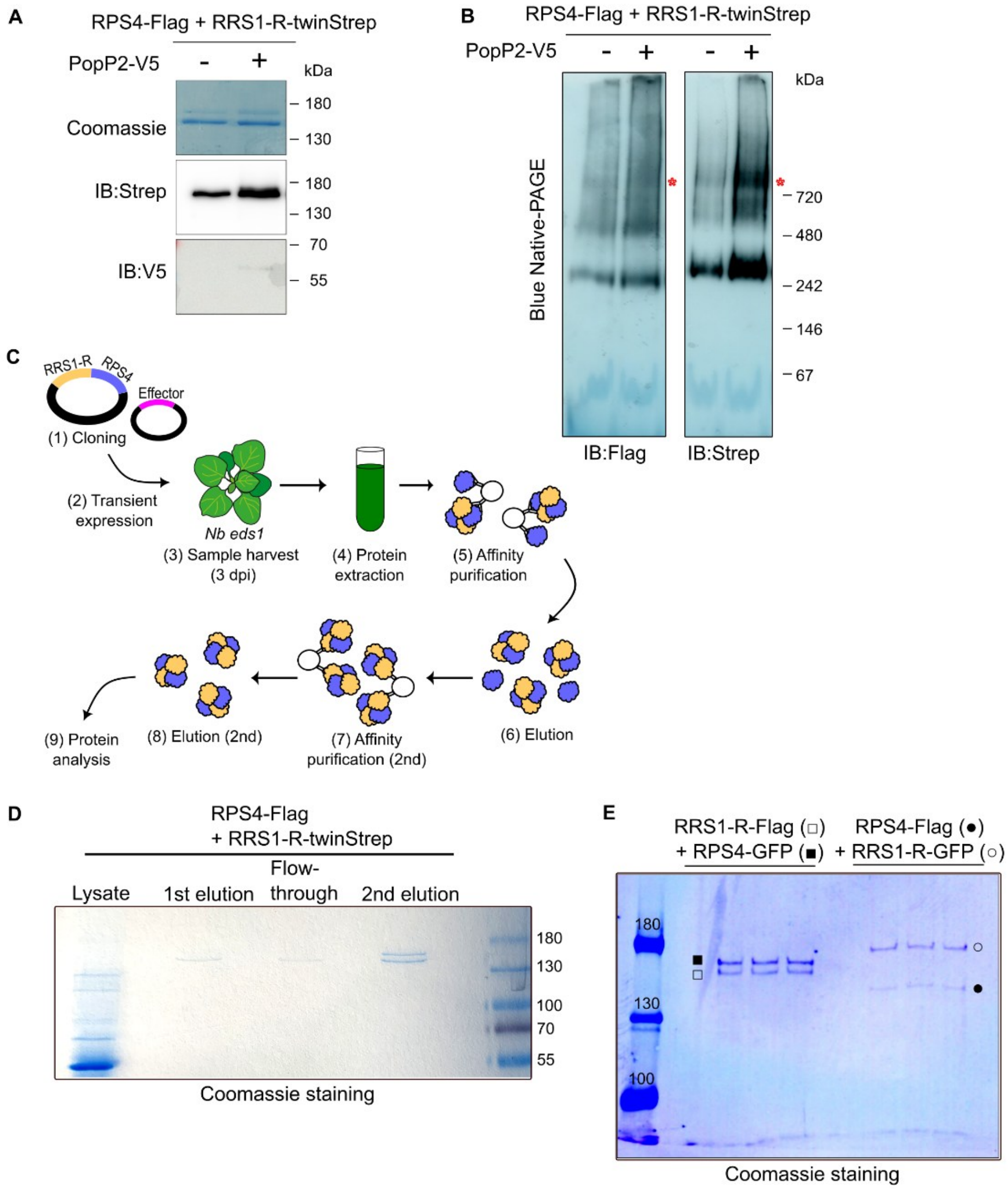

Fig. S1. Purification of RRS1-R/RPS4 complex by transient expression in *N. benthamiana (Nb)*.
A. RPS4-Flag and RRS1-R-twinStrep were co-expressed with or without effector PopP2 in *Nb eds1* and purified using anti-Flag beads. Immunoprecipitated proteins were eluted using 3xFlag peptide, denatured, then subsequently loaded on SDS-PAGE for Coomassie staining (to detect RPS4) or immunoblot (to detect RRS1-R and PopP2). Molecular weights of markers are shown on the right. Similar results were obtained at least three times.
B. RRS1-R-/RPS4 protein complex from A were loaded on BN-PAGE, followed by immunoblotting. Asterisk indicates RRS1-R/RPS4 complex. Molecular weights of markers are shown on the right. Similar results were obtained at least three times.
C. Schematic diagram of tandem-affinity purification from *Nb* or *Nb eds1.*
D,E. Tandem affinity purification of RRS1-R/RPS4 complex. Proteins were extracted from *Nb eds1* (D) or wild-type (E) plants transiently expressing RPS4-Flag with RRS1-R-twinStrep (D), or RPS4 or RRS1-R tagged with Flag or GFP, or vice versa. Protein extracts were immunoprecipitated using anti-Flag beads and eluted with 3xFlag peptide. The eluates were subsequently enriched with Strep-tactin resin (D) or anti-GFP resin (E), and bound protein complex were eluted with 10 mM

biotin (D) or boiled (E). Coomassie staining was performed. Molecular weights of markers are shown on the right. Similar results were obtained at least three times.

Figure S2

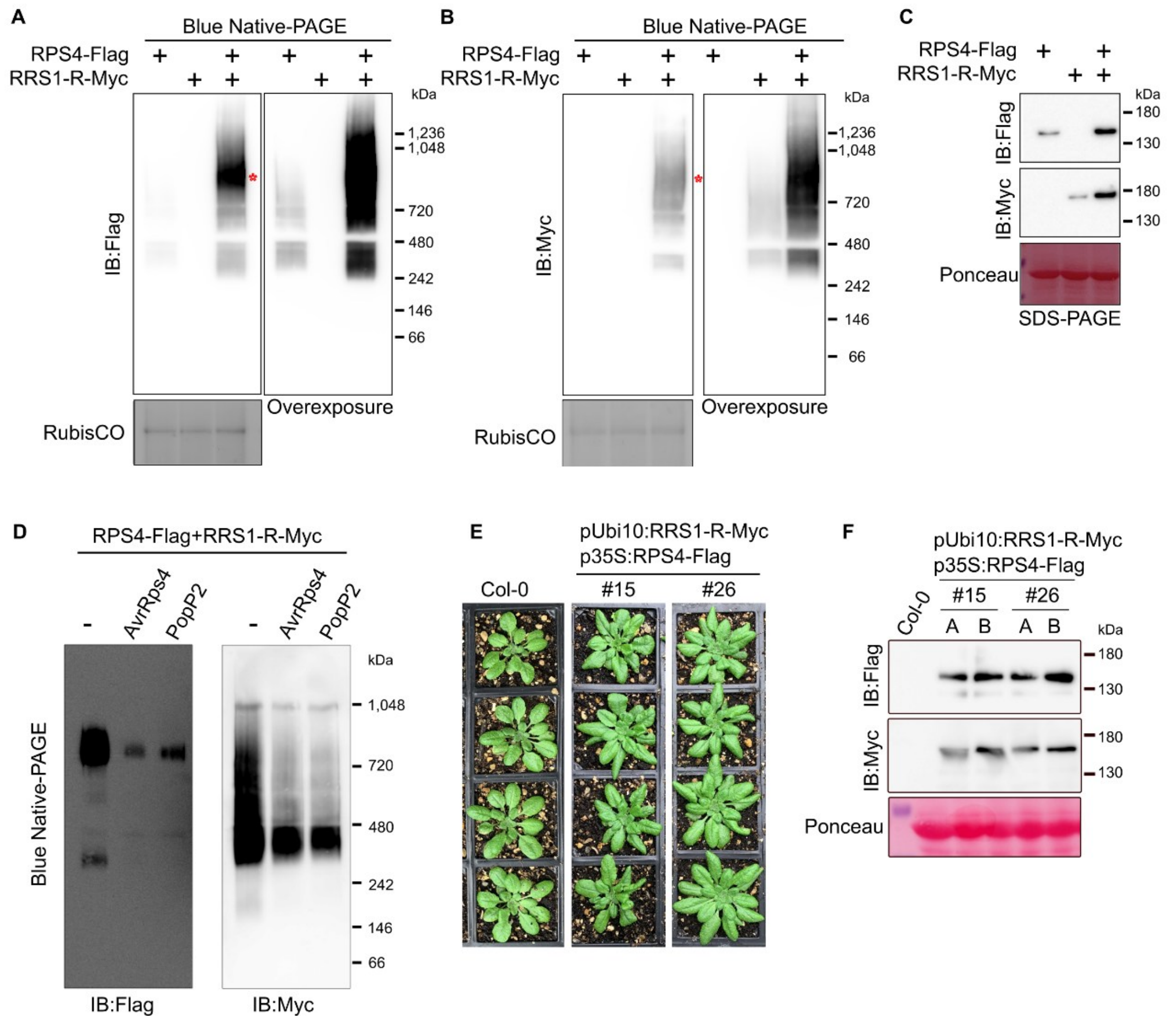

Fig. S2. RRS1-R/RPS4 complex formation in *Nb* and Arabidopsis.

A, B. RPS4 (B) and RRS1-R (C) in *Nb eds1* show slower migration on blue native (BN)-PAGE. Transiently expressed RPS4-Flag and/or RRS1-R-Myc proteins were loaded on BN-PAGE, followed by immunoblotting. RubisCO complex serves as loading control. Asterisks indicate RRS1-R/RPS4 complex. Overexposed images of the same membranes are indicated as 'Overexposure'. Molecular weights of markers are shown on the right. Similar results were obtained at least three times.

C. RRS1-R and RPS4 proteins are stabilized upon co-expression. Samples from A and B were denatured, loaded on SDS-PAGE, followed by immunoblotting. Molecular weights of markers are shown on the right. Ponceau S staining serves as loading control. Similar results were obtained at least three times.

D. RRS1-R and RPS4 were transiently expressed in *Nb eds1* in the presence or absence of effector AvrRps4 and PopP2. Proteins were extracted and aliquot of sample was loaded on BN-PAGE. Molecular weights of markers are shown on the right. Similar results were obtained at least three times.

E. Growth phenotype of RRS1-R-Myc, RPS4-Flag overexpressing Arabidopsis transgenic line. Two lines (#15, #26) overexpressing RRS1-R and RPS4 were chosen and grown for 4 weeks. Col-0 plants serve as control. Similar results were observed at least three independent times.

F. Protein extraction of RRS1-R-Myc, RPS4-Flag overexpressing Arabidopsis transgenic lines. Leaves from two independent plants of each transgenic line were collected from E, and proteins

were extracted, denatured, and loaded on SDS-PAGE. Protein extracted from Col-0 serve as negative control. Ponceau S staining of the SDS-PAGE serve as loading control. Molecular weights of markers are shown on the right.

Figure S3

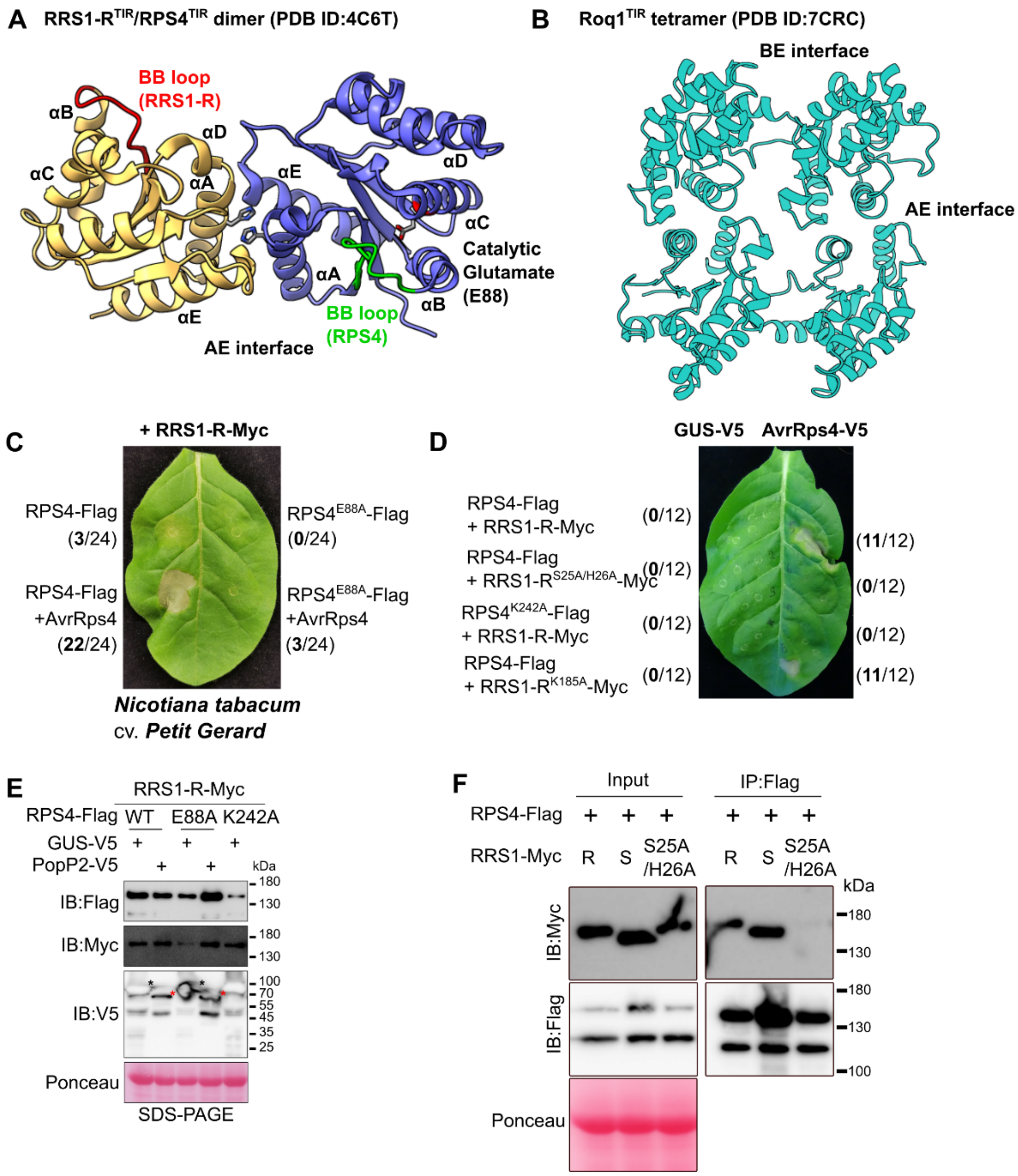

Fig. S3. TIR domain mutations abrogate effector-dependent activation.

A. Illustration of residues and interfaces between TIR^{RRS1} (yellow) and TIR^{RPS4} (purple, PDB ID:4C6T). Position and name of α-helices are shown, along with the histidine rings responsible for AE interface. Catalytic glutamate residue from RPS4 is shown in red. BB loops are highlighted in green (RPS4) and red (RRS1-R).

B. Illustration of activated TIR^{Roq1} tetramer (PDB ID:7CRC). AE interface corresponding to the AE interface in A and BE interface are shown.

C. RPS4 or catalytic mutant (E88A) with RRS1-R-Myc, in the presence or absence of effector were expressed in *N. tabacum* and imaged 5dpi. Number of HR occurrences (in bold) out of total number of leaves tested are indicated in parentheses.

D. AE interface mutation of RRS1-R, P-loop motif mutation of RPS4 and RRS1-R were expressed with corresponding wild-type RRS1-R or RPS4 in the presence or absence of effector AvrRps4. Images were taken 5dpi. Number of HR occurrences (in bold) out of total number of leaves tested are indicated in parentheses.

E. Protein samples from main Figure 3 A and B were denatured and loaded on SDS-PAGE for immunoblotting. Ponceau S staining of the SDS-PAGE serve as loading control. Molecular weights of markers are shown on the right.

F. Co-immunoprecipitation of RRS1-R, RRS1-S and RRS1-R AE mutant by RPS4-Flag. Proteins were expressed in *Nb eds1*, proteins were extracted and immunoprecipitated with anti-Flag beads. Eluted samples were loaded on SDS-PAGE and detected with immunoblots. Ponceau S staining of the SDS-PAGE serve as loading control. Molecular weights of markers are shown on the right.

Figure S4

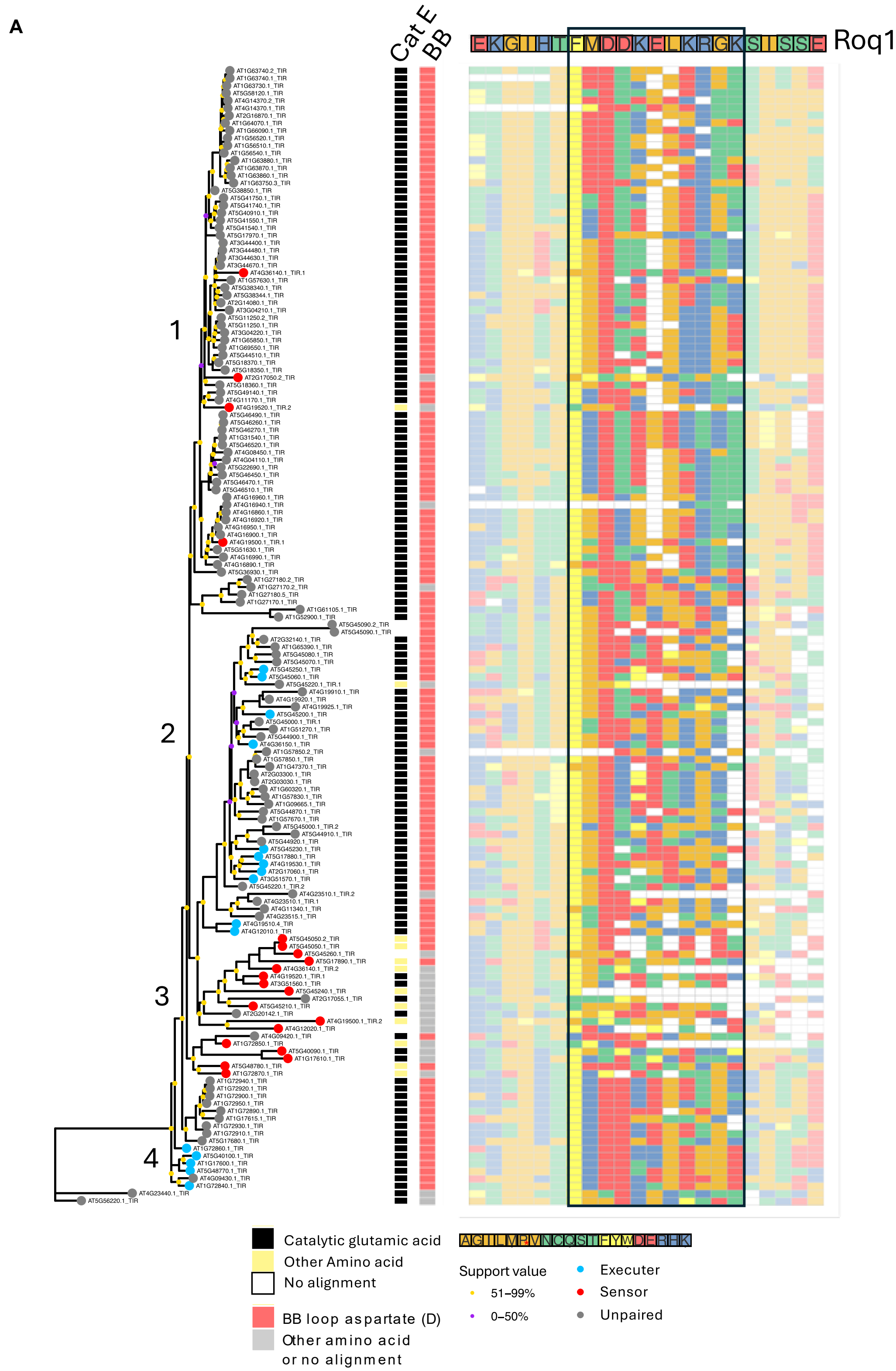

Fig. S4. Phylogenetic tree of TIR domains identified in Col-0 reference genome of Arabidopsis. 152 unique TIR domains from Col-0 reference genome were selected. Amino acids in the corresponding

structural positions to the Roq1 catalytic glutamate (E86), conserved BB-loop aspartate (D45), the BB-loop (F43-K53) and surrounding region (E37-E58) were extracted from alignments and shown. The tree was rooted to divergent TIR-N proteins AT5G56220.1 and AT4G23440.1.

Figure S5

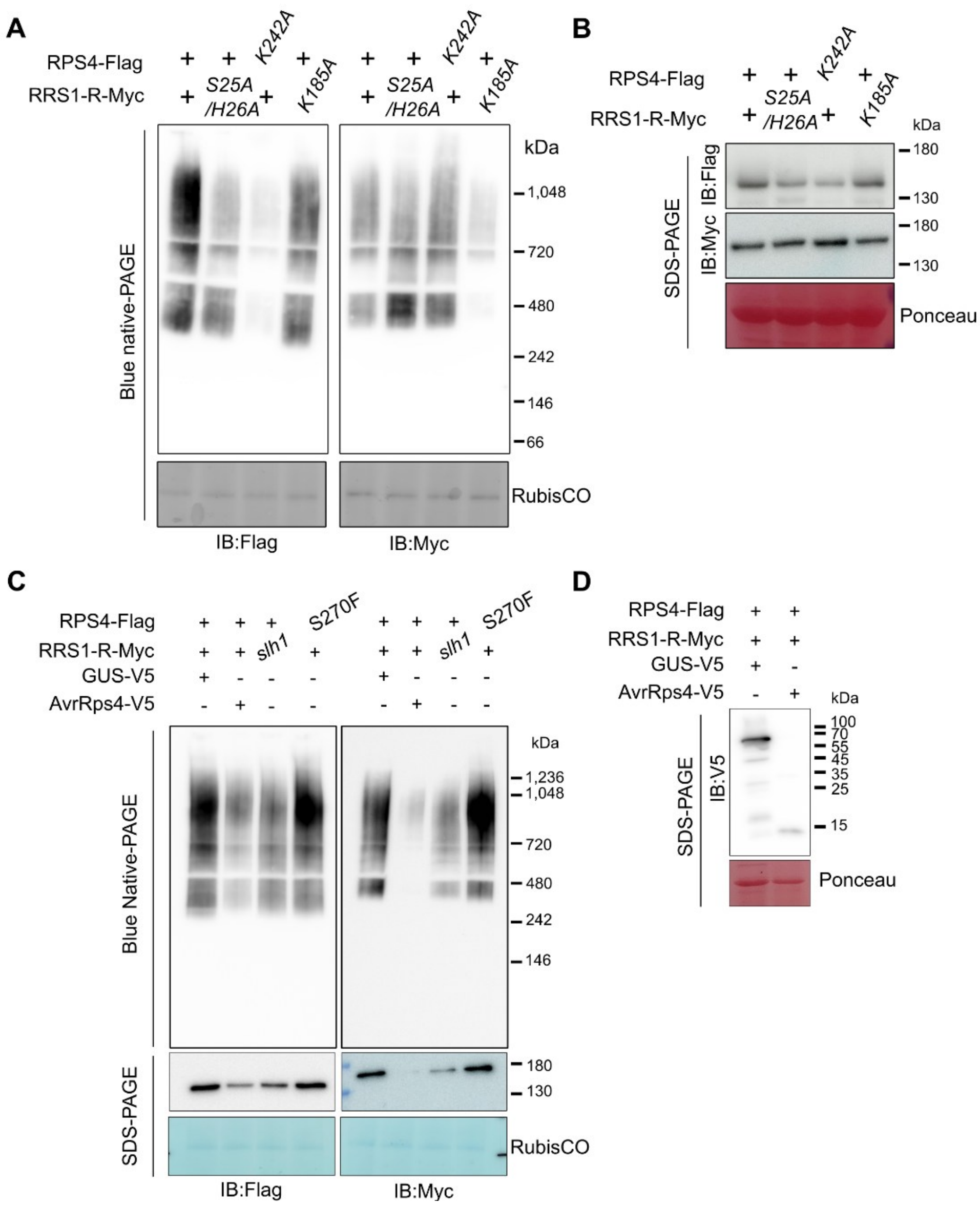

Fig. S5. Effect of RRS1-R/RPS4 complex formation by P-loop mutations and autoactive mutations. A, B. RPS4 and RRS1-R and its P-loop mutant derivatives transiently expressed in *Nb eds1* were loaded on BN-PAGE, followed by immunoblotting (A). Same samples were denatured and loaded on SDS-PAGE followed by immunoblotting (B). RubisCO complex for BN-PAGE, and Ponceau S staining for SDS-PAGE, serves as loading control. Molecular markers are shown on right. Similar results were obtained at least three times.

C. RPS4 and RRS1-R and its autoactive mutant derivatives transiently expressed in *Nb eds1* were loaded on BN-PAGE, followed by immunoblotting. Same samples were denatured, loaded on SDS-PAGE followed by immunoblotting. RubisCO for BN-PAGE, and Ponceau S staining for SDS-PAGE, serves as loading control. Molecular markers are shown on right. Similar results were obtained at least three times.

D. Samples expressing RPS4, RRS1-R with GUS-V5 or AvrRps4-V5 from C was immunoblotted with anti-V5. Ponceau S staining serve as loading control. Molecular markers are shown on right. Similar results were obtained at least three times.

Figure S6

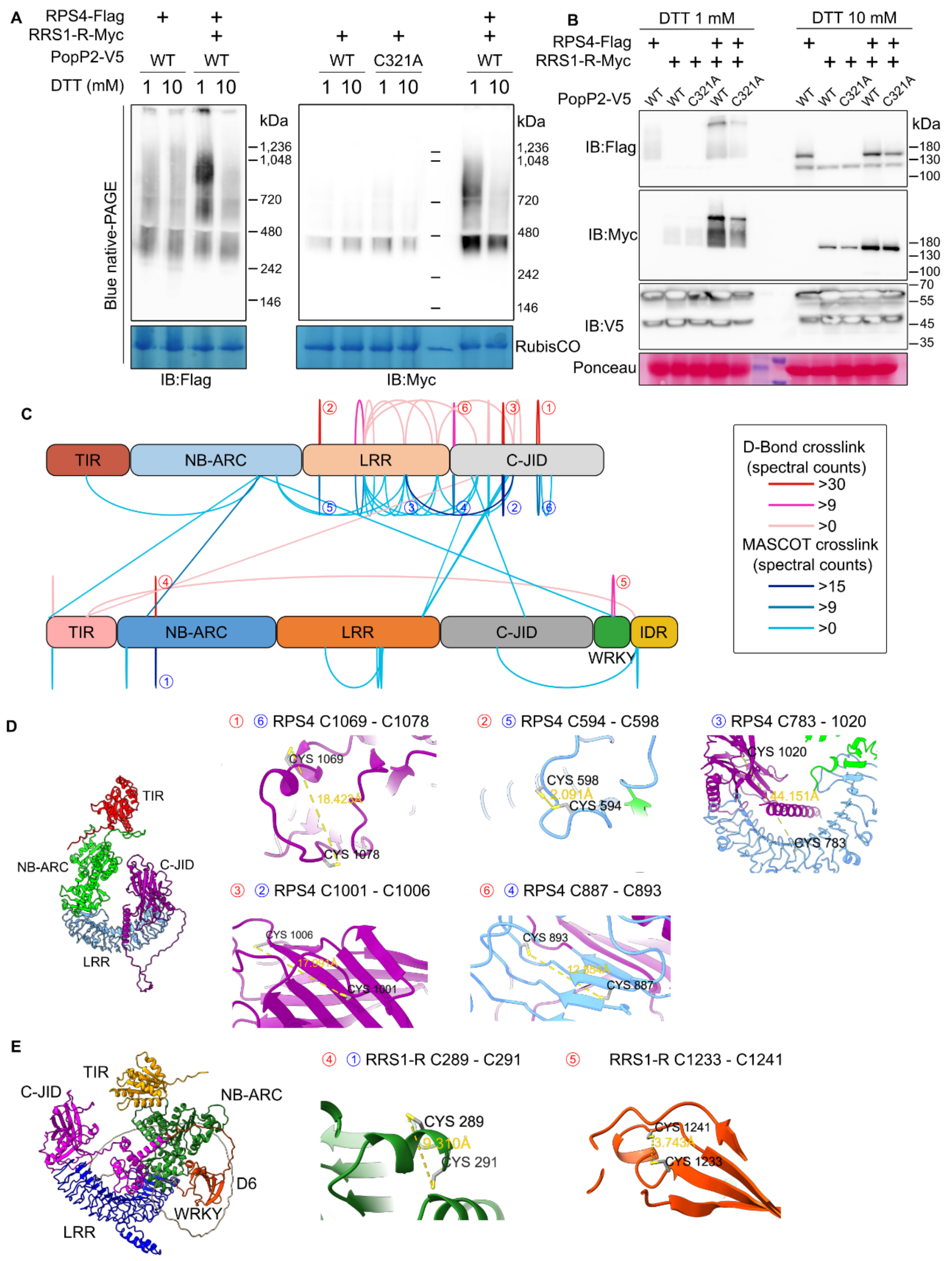

Fig. S6. Low-reducing conditions are required to maintain RRS1-R/RPS4 complex assembly.
A. RRS1-R/RPS4 complex dissociate under high reducing conditions. RRS1-R and RPS4 transiently expressed with PopP2 (wild-type or mutant C321A) in *Nb eds1* leaves were extracted with high (10 mM DTT) or low (1 mM DTT) reducing conditions, loaded on BN-PAGE, followed by

immunoblotting. RubisCO complex observed on blue native-PAGE gel serves as loading control. Molecular weights of markers are shown on the right of each panel. Similar results were obtained at least three times.

B. Protein extracts from A were incubated with SDS only, and loaded on SDS-PAGE, followed by immunoblotting. Ponceau S staining of the SDS-PAGE serve as loading control. Molecular weights of markers are shown on the right. Similar results were obtained at least three times.

C. Schematic diagram of the identified disulfide bonds using MASCOT (blue) and D-Bond (red) analysis. Numbers in circle for bonds show the top 6 most abundant disulfide bonds from each analysis.

D. Prediction of disulfide bond formation and their predicted distance in the RPS4 model from Alphafold3. Numbers from C are shown next to the identified disulfide bonds. Distance between the sulfur atom from the corresponding cysteine residue is shown in yellow.

E. Prediction of disulfide bond formation and their predicted distance in the RRS1-R model from Alphafold3. Numbers from C are shown next to the identified disulfide bonds. Distance between the sulfur atom from the corresponding cysteine residue is shown in yellow.

Figure S7

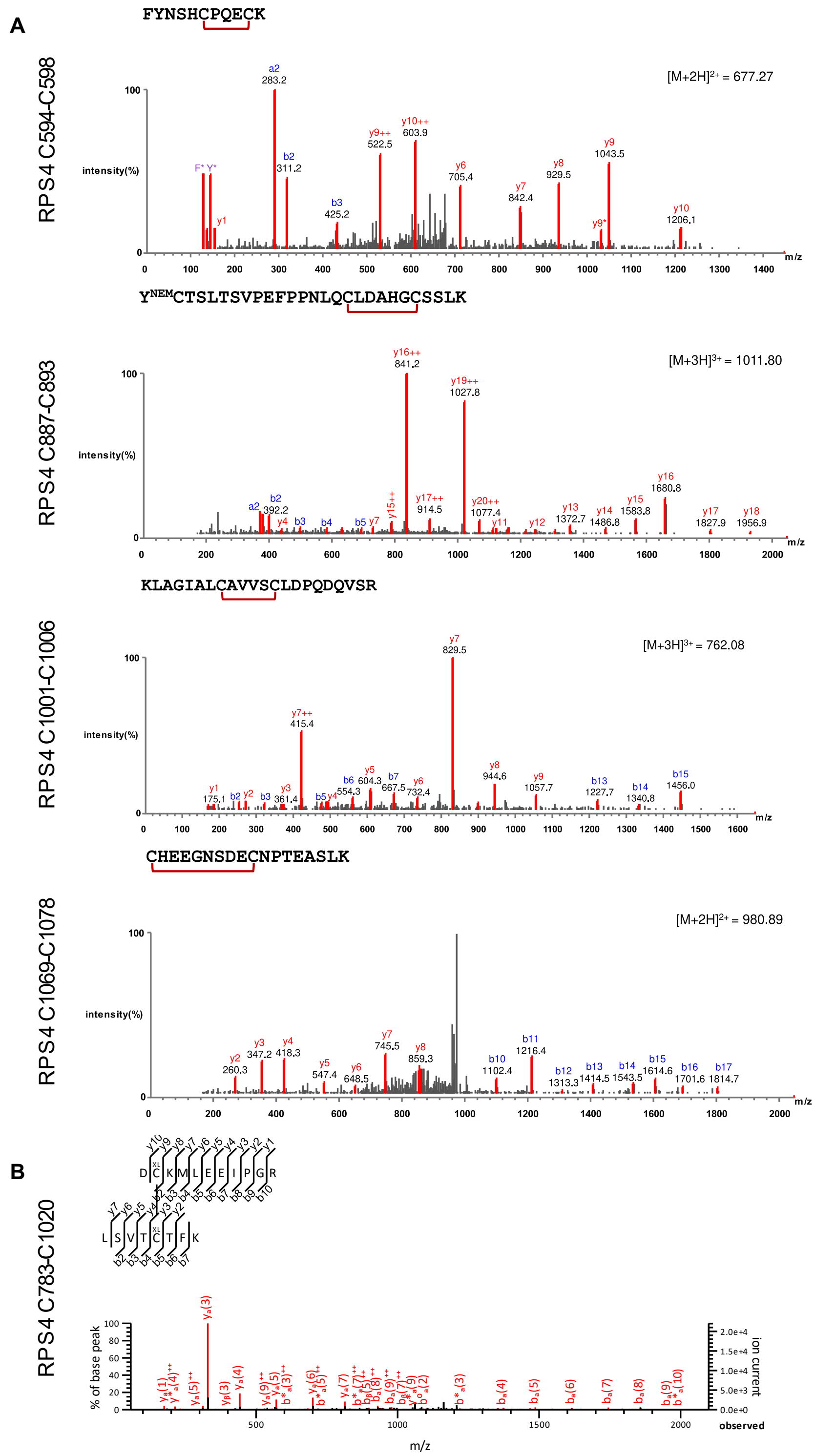

Fig. S7. Spectrum data of identified disulfide bonds from RPS4.
A. Spectrum data from D-Bond analysis for the crosslinked peptides by disulfide bonds.
B. Spectrum data from Mascot analysis for the crosslinked peptides by disulfide bonds.

Figure S8

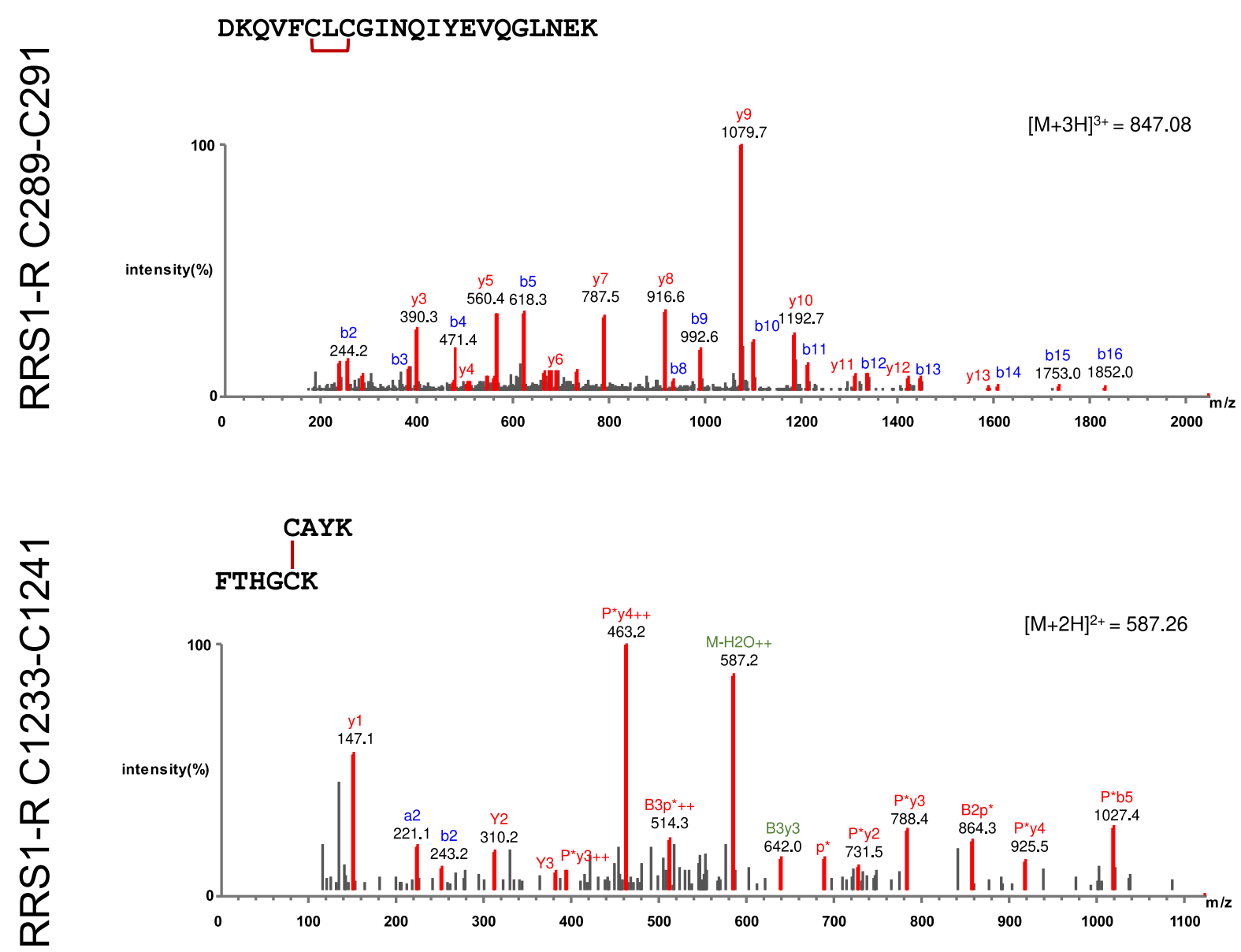

Fig. S8. Spectrum data of identified disulfide bonds from RPS4.
Spectrum data from D-Bond analysis for the crosslinked peptides by disulfide bonds.

Figure S9

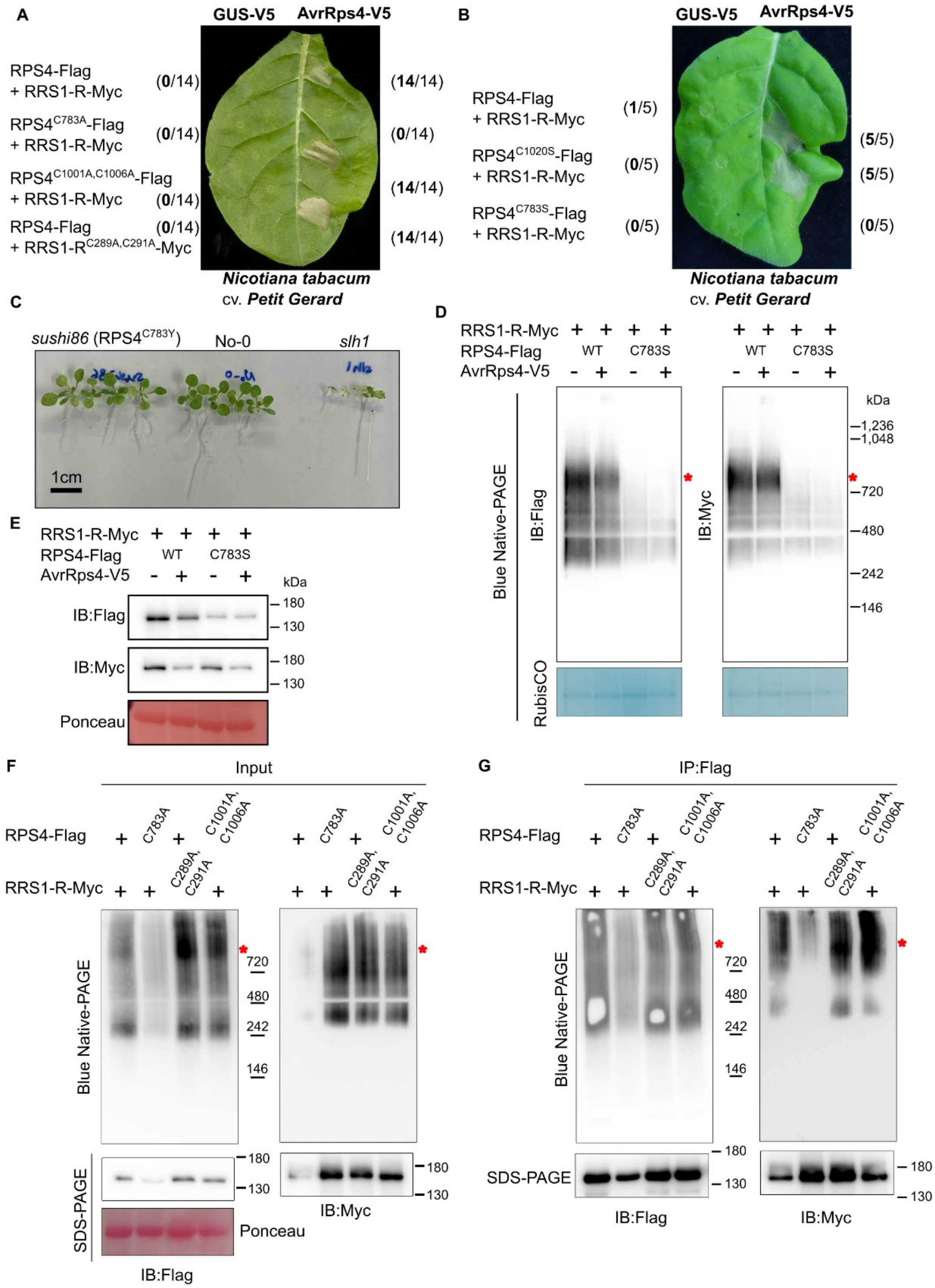

Fig. S9. RPS4 C783 is required for stability of RPS4 and RRS1-R/RPS4 complex formation. A. Cysteine residues of RPS4 or RRS1-R identified to form disulfide bonds were mutated to alanine, and tested for effector-dependent activation of HR. After transient infiltration of wild-type of mutant RPS4-Flag and RRS1-R-Myc proteins with or without AvrRps4-V5 into *N. tabacum* cv. *Petit Gerard*

leaves, images were taken 5 dpi. Number of HR occurrences (in bold) out of total number of leaves tested are indicated in parentheses.

B. C783 and C1020 residues of RPS4 identified to form disulfide bonds were mutated to serine (S), and tested for effector-dependent activation of HR. After transient infiltration of wild-type of mutant RPS4-Flag and RRS1-R-Myc proteins with or without AvrRps4-V5 into *N. tabacum* cv. *Petit Gerard* leaves, images were taken 5 dpi. Number of HR occurrences (in bold) out of total number of leaves tested are indicated in parentheses.

C. Arabidopsis *sushi86* ($RPS4^{C783Y}$), No-0 and *slh1* seedlings were germinated on ½ MS media with 1% sucrose and grown for 10 days.

D. Slower migrating forms of RRS1-R and RPS4 are lost in $RPS4^{C783S}$ mutant. Proteins were extracted from *Nb eds1* leaves expressing RPS4-Flag and its $RPS4^{C783S}$ mutant with RRS1-R-Myc in the presence or absence of effector AvrRps4. Extracts were loaded on BN-PAGE, followed by immunoblotting. Asterisk indicate RRS1-R/RPS4 complex. RubisCO complex serves as loading control. Molecular weights of markers are shown on the right. Similar results were observed three times.

E. Protein accumulation of $RPS4^{C783S}$ is reduced compared to wild-type RPS4. Extracts from C were denatured and loaded on SDS-PAGE, followed by immunoblotting. Ponceau S staining of the SDS-PAGE serve as loading control. Molecular weights of markers are shown on the right. Similar results were observed three times and representative images are shown.

F. RPS4 and RRS1-R and its cysteine mutant derivatives transiently expressed in *Nb eds1* were loaded on BN-PAGE or SDS-PAGE, followed by immunoblotting. Asterisk indicate RRS1-R/RPS4 complex. Molecular weights of markers are shown on the right of the panel. Ponceau S staining serve as loading control. Similar results were obtained at least three times.

G. Protein extracts from F were immunoprecipitated with anti-Flag beads. Proteins were eluted with 3xFlag peptide, and eluates were loaded on BN-PAGE, followed by immunoblotting. Asterisk indicate RRS1-RPS4 complex. Molecular weights of markers are shown on the right of the panel. Ponceau S staining serve as loading control. Similar results were obtained at least three times.

Figure S10

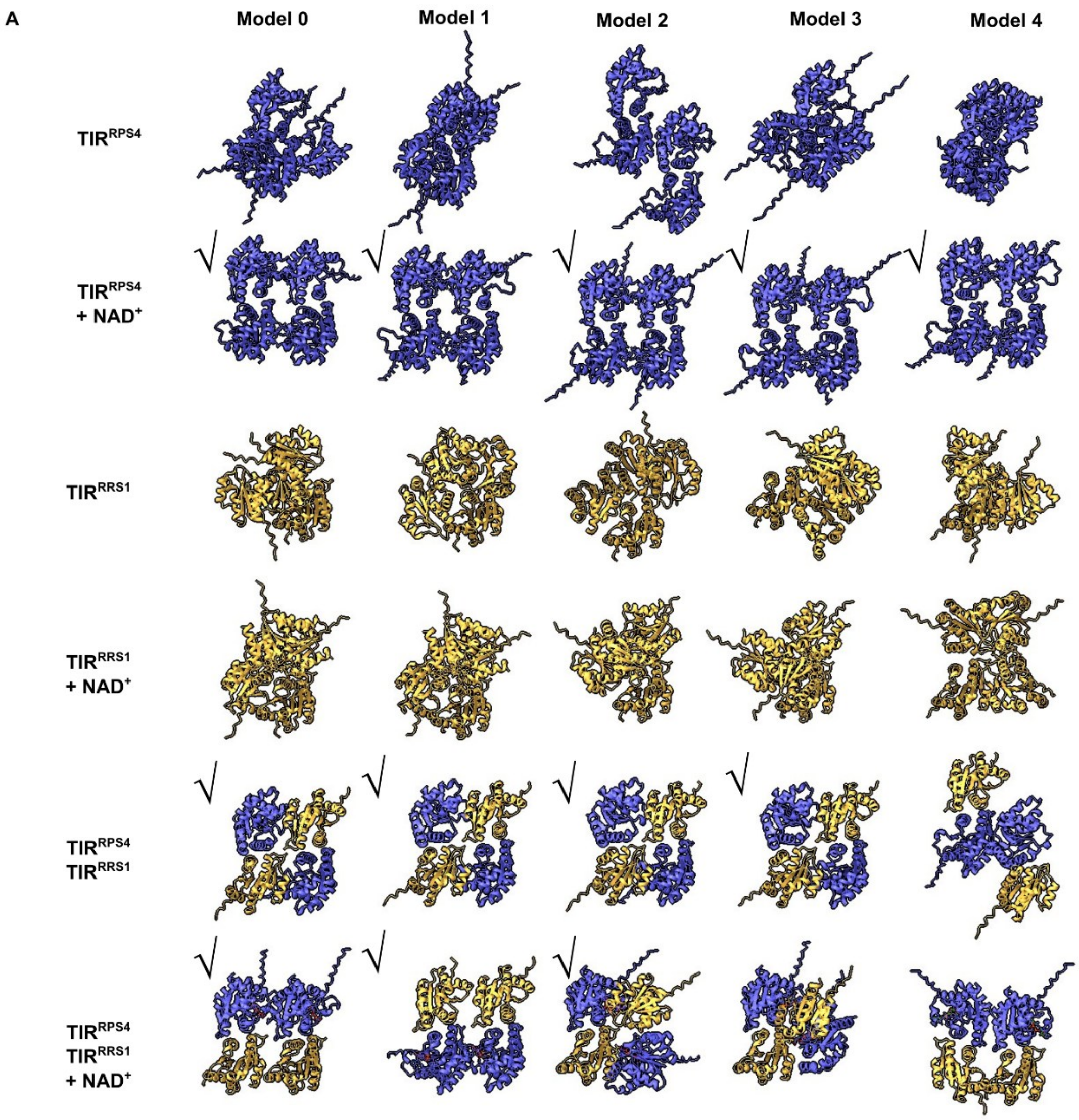

B

	PTM					IPTM				
TIR tetramer models	model 0	model 1	model 2	model 3	model 4	model 0	model 1	model 2	model 3	model 4
TIR^{RPS4}	0.38	0.38	0.35	0.34	0.34	0.21	0.21	0.17	0.16	0.16
TIR^{RPS4} + NAD^+	0.83	0.83	0.83	0.83	0.83	0.81	0.81	0.81	0.81	0.81
TIR^{RRS1}	0.30	0.30	0.3	0.3	0.3	0.11	0.1	0.1	0.1	0.1
TIR^{RRS1}+NAD^+	0.36	0.34	0.33	0.32	0.31	0.2	0.18	0.17	0.17	0.16
TIR^{RPS4} + TIR^{RRS1}	0.46	0.46	0.46	0.44	0.41	0.32	0.32	0.32	0.3	0.26
TIR^{RPS4}+TIR^{RRS1}+NAD^+	0.44	0.43	0.42	0.43	0.41	0.31	0.3	0.3	0.3	0.28

Fig. S10. Tetramer models of TIR^{RPS4} and TIR^{RRS1} in the presence or absence of substrate.
A. Alphafold3 models of TIR^{RPS4}, TIR^{RPS4} with NAD^+, TIR^{RRS1}, TIR^{RRS1} with NAD^+, TIR^{RPS4} and TIR^{RRS1} domains, and TIR^{RPS4} and TIR^{RRS1} with NAD^+. TIR^{RPS4}; purple, and TIR^{RRS1}; yellow, number of Alphafold3 models assuming a ROQ1 TIR-like assembly indicated with √ mark. Figures prepared using ChimeraX, models.
B. ipTM (interface predicted temple modelling score) and pTM (predicted template modelling score) values, generated using Alphafold3, are shown in a table for each model.

Dataset S1A. 152 Arabidopsis TIR domain sequences for Figure S4.

Dataset S1B. Disulfide bonds identified through MASCOT analysis.

Dataset S1C. Disulfide bonds identified through D-bond analysis.

Dataset S1D. Organisms and genetically modified organisms generated used in this study.

Dataset S1E. Constructs used in this study.

SI References

1. P. Jones *et al.*, InterProScan 5: genome-scale protein function classification. *Bioinformatics* **30**, 1236-1240 (2014).
2. M. A. Larkin *et al.*, Clustal W and Clustal X version 2.0. *Bioinformatics* **23**, 2947-2948 (2007).
3. S. Capella-Gutierrez, J. M. Silla-Martinez, T. Gabaldon, trimAl: a tool for automated alignment trimming in large-scale phylogenetic analyses. *Bioinformatics* **25**, 1972-1973 (2009).
4. B. Q. Minh *et al.*, IQ-TREE 2: New Models and Efficient Methods for Phylogenetic Inference in the Genomic Era. *Mol Biol Evol* **37**, 1530-1534 (2020).
5. J. Jumper *et al.*, Highly accurate protein structure prediction with AlphaFold. *Nature* **596**, 583-589 (2021).
6. S. B. Pandit, J. Skolnick, Fr-TM-align: a new protein structural alignment method based on fragment alignments and the TM-score. *BMC Bioinformatics* **9**, 531 (2008).
7. E. Paradis, J. Claude, K. Strimmer, APE: Analyses of Phylogenetics and Evolution in R language. *Bioinformatics* **20**, 289-290 (2004).
8. R. A. M. Villanueva, Z. J. Chen, ggplot2: Elegant Graphics for Data Analysis (2nd ed.). *Measurement: Interdisciplinary Research and Perspectives* **17**, 160 - 167 (2019).
9. G. Yu, D. K. Smith, H. Zhu, Y. Guan, T. T.-Y. Lam, ggtree: an r package for visualization and annotation of phylogenetic trees with their covariates and other associated data. *Methods in Ecology and Evolution* **8**, 28-36 (2017).
10. L. Zhou *et al.*, ggmsa: a visual exploration tool for multiple sequence alignment and associated data. *Brief Bioinform* **23** (2022).
11. E. v. d. B. Campitelli, Teun; olivroy (2020) ggnewscale: Multiple Fill and Color Scales in ggplot2 (v.0.5.0). (Zenodo).
12. Y. Perez-Riverol *et al.*, The PRIDE database at 20 years: 2025 update. *Nucleic Acids Res* **53**, D543-D553 (2025).
13. A. T. Kong, F. V. Leprevost, D. M. Avtonomov, D. Mellacheruvu, A. I. Nesvizhskii, MSFragger: ultrafast and comprehensive peptide identification in mass spectrometry-based proteomics. *Nat Methods* **14**, 513-520 (2017).
14. J. E. Elias, S. P. Gygi, Target-decoy search strategy for increased confidence in large-scale protein identifications by mass spectrometry. *Nat Methods* **4**, 207-214 (2007).
15. S. Choi *et al.*, New algorithm for the identification of intact disulfide linkages based on fragmentation characteristics in tandem mass spectra. *J Proteome Res* **9**, 626-635 (2010).
16. S. Na, J. Kim, E. Paek, MODplus: Robust and Unrestrictive Identification of Post-Translational Modifications Using Mass Spectrometry. *Anal Chem* **91**, 11324-11333 (2019).
17. D. M. Creasy, J. S. Cottrell, Unimod: Protein modifications for mass spectrometry. *Proteomics* **4**, 1534-1536 (2004).
18. J. Abramson *et al.*, Accurate structure prediction of biomolecular interactions with AlphaFold 3. *Nature* **630**, 493-500 (2024).
19. E. C. Meng *et al.*, UCSF ChimeraX: Tools for structure building and analysis. *Protein Sci* **32**, e4792 (2023).
